# Three-phase extraction of polysaccharide from *Stropharia rugosoannulata*: Process optimization, structural characterization and bioactivities

**DOI:** 10.3389/fimmu.2022.994706

**Published:** 2023-01-11

**Authors:** Xinxin Li, Zhiqiang Zhang, Li Wang, Haoqiang Zhao, Yahui Jia, Xia Ma, Jinzhan Li, Yi Wang, Bingji Ma

**Affiliations:** ^1^ Department of Traditional Chinese Medicine, Henan Agricultural University, Zhengzhou, China; ^2^ School of Pharmacy, Henan University of Chinese Medicine, Zhengzhou, China; ^3^ College of Animal medcine, Henan University of Animal husbandry and Economy, Zhengzhou, China; ^4^ Henan Jinlong Mushroom Industry Co. LTD, Shangqiu, China; ^5^ Business Development, GeneGenieDx Corporation, San Jose, CA, United States

**Keywords:** *Stropharia rugosoannulata*, polysaccharide, three-phase extraction, structure, bioactivities

## Abstract

The isolation of *Stropharia rugosoannulata* polysaccharide (SRP) by three-phase extraction was optimized, and its structure and biological activities were identified. The optimal extraction conditions were: mass fraction of ammonium sulfate, 20%; volume ratio of sample solution to t-butanol, 1:1.5; extraction temperature, 35°C. Under these conditions, the yield of SRP was 6.85% ± 0.13%. SRP was found to be composed of glucose (35.79%), galactose (26.80%), glucuronic acid (9.92%), fructose (8.65%), xylose (7.92%), fucose (4.19%), arabinose (3.46%) and rhamnose (3.26%), with the molecular weight of 27.52 kDa. The results of DPPH, hydroxyl, ABTS^+^ radical scavenging and reducing power tests showed that SRP had good antioxidant capacities. SRP had no cytotoxic effect on RAW264.7 macrophages at the concentrations of 25-200 μg/mL, and could significantly promote phagocytosis activity and cell migration according to CCK-8 assay, phagocytosis assay and cell scratch experiment. SRP can significantly stimulate the transcript expression levels of TNF-α, IL-1β and IL-6, as determined by RT-PCR and Western blot assays. SRP activated the TLR4/NF-κB signaling pathway, and autophagy also occurred. These results suggest that SRP is a safe antioxidant and immunomodulator, and that it can be used in the development of functional foods and/or pharmaceuticals.

## 1 Introduction

*Stropharia rugosoannulata* Farlow (SR) is a mushroom, also known as wine cap stropharia. It is one of the mushrooms recommended by the Food and Agriculture Organization of the United Nations (FAO) for cultivating, so it is now grown in many developing countries (1). The fruiting body of SR has been reported to include multiple beneficial components, such as flavonoids, vitamins, polysaccharides, and protein. Thus it has potential medicinal value (2).

Fungal polysaccharides have been isolated from fruiting bodies or from fungal mycelia in fermentation broth. Researches have demonstrated that fungal polysaccharides have anti-virus (3), anti-tumor (4), immunomodulatory and anti-aging activities (5). Many have already been developed into a variety of drugs and functional food additives (6). Recently, fungal polysaccharides and their complexes have attracted more attention. *S. rugosoannulata* polysaccharide is one of many fungal polysaccharides and its medicinal value has been gradually recognized. Many studies have shown that polysaccharides from mushrooms have immunomodulatory activity and are good natural immunomodulatory adjuvants (3, 5).

The method of extracting polysaccharides from fungal mycelia affects not only yield but also the structure and biological activities of the polysaccharides extracted. At present, the conventional extraction method is water extraction followed by ethanol precipitation (7). This method has the shortcomings of long time and low efficiency. Three-phase extraction is a new method to extract active ingredients. It has the advantages of high efficiency and environmental sustainability. Recently, this method has been applied to extract polysaccharides from animal material (e.g., shrimp shell (8); *Corbicula fluminea* (9)), plant material (e.g., aloe (10)), and microorganisms. However, there are few reports of using it to isolate polysaccharides from edible or medicinal fungi.

In this research, the polysaccharides of *S. rugosoannulata* were extracted by three-phase extraction. Physicochemical properties such as molecular weight and monosaccharide composition were determined. Scanning electron microscopy (SEM) was used for morphological studies. Antioxidant and immunomodulatory properties were evaluated comprehensively through *in vitro* antioxidant experiments and cell models. The results provide information for the development of SRP as a functional component in foods or medicine.

## 2 Materials and methods

### 2.1 Reagents

*S. rugosoannulata* was collected from a field in Yucheng County, Henan Province, China. Trypsin and RPMI1640 were purchased from Gibco (Grand Island, NY, United States). The Cell counting kit-8 (CCK-8) was purchased from Han Heng Biotechnology Co., Ltd. (Shanghai, China). Lipopolysaccharide (LPS) was purchased from Sigma Chemical Co. (St. Louis, MO, USA). The remaining chemicals or reagents were all of laboratory or analytical quality.

### 2.2 Extraction and preparation of SRP

Referring to the method of Yan (9), we made minor modifications. The fruiting body of SR was dried at 45°C in the air-drying box and then ground into powder with a grinder. Then, 95% ethanol (1:200, g/mL) was refluenced 5 hours, repeated 3 times, which was designed to skim and decolorize. Distilled water was added according to the ratio of 1:30 (w/v), and extracted by stirring in a water bath at 100°C for 2 hours. After continuous extraction for two times, the filtrate was merged for two times and concentrated. Added 15-35% (NH_4_)_2_SO_4_ (w/v) to SR powder and vortexed gently. The suspension was then diluted with t-butanol at a ratio of 1.5:1 to 1:2.5 (v/v). Kept the combination at 20-40°C for an hour. Then, the mixture was placed in a centrifuge at 4000 RPM/separation center for 10 minutes to accelerate three-phase extraction. After centrifugation, the three phases formed were carefully separated. The lower phase is mainly ammonium sulfate and polysaccharides, and polysaccharides were freeze-dried after removing inorganic salts by molecular dialysis (3500 Da).

### 2.3 Structural characterization and molecular morphology

#### 2.3.1 Physicochemical characterizations of SRP

The basic components of carbohydrate, protein and polyphenol were detected according to our previous methods (11, 12).

#### 2.3.2 Molecular weight determination

Gel permeability chromatography (GPC) was used to determine the molecular weight distribution of SRP using a Sugar KS805 column fitted with an Agilent refractive index detector.

#### 2.3.3 Studies on the composition of monosaccharides

Using high-performance anion exchange chromatography (HPAEC) coupled with a pulse ammeter detector, we determined the monosaccharide composition of SRP. Gas chromatography (GC) was used to determine the monosaccharide composition of SRP following the procedure described by Yang (13). 5 mg of SRP was dissolved in 1mL 2.5 M trifluoroacetic, and was mixed by the vortex. The sample was placed in the oven for 1.5 hours at 121°C, during which the sample was vortexed every 30 minutes. Once the SRP were hydrolyzed, they were diluted, filtered, and injected into an HPAEC system (Dionex, ICS-5000+, USA) using an ASAP automatic sampler and a calcium carbonate PA-20 column (3×150 mm, Dionex). The monosaccharides in SRP were determined by eight different monosaccharides.

#### 2.3.4 X-ray diffraction test

Empyrean X-ray diffractometer (panalit LTD., Netherlands) was used to record the diffraction patterns of SRP at 40kV and 15 mA. The scanning speed was 3°/min, the step size was 0.01, and the 2 range was 2 to 40°.

#### 2.3.5 SEM analysis

An SEM Model S-4800 II FESEM (Hitachi, Japan) was used to study the molecular morphologies of SRP. A layer of gold foil was laid on the sample and then placed on the substrate. Under a high vacuum and 10.0 kV voltage, the image was observed by a magnification of 100× or 500× fold.

### 2.4 Antioxidant activities analysis *in vitro* of SRP

#### 2.4.1 Analysis of DPPH free radical scavenging

The DPPH radicals scavenging activity of SRP was determined according to the method of Wang et al. (11). 500 μL different concentrations of SRP (0.05, 0.1, 0.5, 1, 2, 4, 6, 8, 10 mg/mL) were mixed with the newly configured 2-diphenyl-1-picrylhydrazyl (DPPH) solution (dissolved in ethanol) in equal volume, and the reaction solution was left in a dark place at 26°C for half an hour. The blank group consisted of the same volume of distilled water as the sample solution, and the positive control had the same concentration of Vc. The absorbance of the reaction mixture was determined at 517 nm. Scavenging activity (percent) against DPPH was calculated by using the following equation:


$$\text{Scavenging ability }\left(\%\right)\;=\;\left[1-\left({\text{A}}_{\text{X}}–{\text{A}}_{\text{X}0}\right)/{\text{A}}_{0}\right]\times 100$$

Where A_0_ was the absorbance of the blank control; A_X_ was the absorbance of the sample solution; A_X0_ was the background absorbance of the sample solution.

#### 2.4.2 Analysis of hydroxyl free radical scavenging

Added 400 μL FeSO_4_ solution to 400 μL different concentrations of SRP (0.05, 0.1, 0.5, 1, 2, 4, 6, 8, 10 mg/mL). After adding 800 μL of H_2_O_2_ solution, the reaction was started and the solution was left to react at 37°C for 30 minutes. The blank group consisted of the same amount of distilled water that was used to replace the sample solution as the positive control had the same concentration of Vc. The 510 nm wavelength of the reaction solution was measured. To determine the hydroxyl scavenging capacity (in percent), the following equation was used:


$$\text{Scavenging ability }\left(\%\right)\;=\;\left[1-\left({\text{A}}_{\text{X}}–{\text{A}}_{\text{X}0}\right)/{\text{A}}_{0}\right]\times 100$$

Where A_0_ was the absorbance of the blank control; A_X_ was the absorbance of the sample solution; A_X0_ was the background absorbance of the sample solution.

#### 2.4.3 Analysis of ABTS^+^ free radical scavenging

ABTS^+^ radicals removing capacity of SRP was according to the work of Qiu et al. (14). The ABTS^+^ working solution was made by reacting 7 mmol/L of ABTS^+^ solution with 2.45 mmol/L of K_2_S_2_O_8_ solution at 26°C for 16 hours in the dark. When using, adjust the absorbance of the ABTS^+^ working solution at 734 nm. Then, took 100 μL different concentrations of SRP (0.05, 0.5, 0.1, 0.5, 1, 2, 4, 6, 8, 10 mg/mL), added 0.6 mL ABTS^+^ solution, mixed evenly and placed at 26°C for 10 minutes. The same concentration of Vc solution as the positive group. The reaction solution’s absorbance at 734 nm was measured. Using these formulae, we were able to determine the ABTS^+^ scavenging efficiency (in percentage):


$$\text{Scavenging ability }\left(\%\right)\;=\;\left[1-\left({\text{A}}_{\text{X}}–{\text{A}}_{\text{X}0}\right)/{\text{A}}_{0}\right]\times 100$$

Where A_0_ was the absorbance of the blank control; A_X_ was the absorbance of the sample solution; A_X0_ was the background absorbance of the sample solution.

#### 2.4.4 Analysis of reducing ability

The method of Gao et al. (15) was used for sample reduction force detection, and some modifications were made. In brief, 200 μL different concentrations of SRP (0.05, 0.1, 0.5, 1, 2, 4, 6, 8, 10 mg/mL) were taken and 500 μL PBS and 100 μL 1% (w/v) iron oxide clock solution was added, then the reaction system was incubated in 50°C for 20 minutes. After quick cooling, it was combined with a 500 μL 10 percent (w/v) trichloroacetic acid solution, and then centrifuged. Next, took 500 μL supernatant, added 500 μL distilled water and 100 μL 0.1% (w/v) FeCl_3_ solution successively, then mixed them evenly at 26°C. The absorbance of the reaction solution was measured at 700 nm and a standard solution with the same quantity of Vc was employed as the positive control. The sample’s reducing power is proportional to its absorbance value, which measures the reaction system’s efficiency.

### 2.5 Immunomodulatory activity assay of SRP

#### 2.5.1 Cell culture

RAW 264.7 macrophages were grown in an incubator with 5% CO_2_ at 37°C in RPMI1640 media supplemented with 10% fetal bovine serum, 100 U/mL penicillin, and 100 g/mL streptomycin. Every 2-3 days, new media will be added.

#### 2.5.2 Cell viability

The cell viability of SRP was detected by the CCK-8 method (16). RAW 264.7 macrophages were placed onto a 96-well plate with 100 μL/well cell suspension and cultured in the cell incubator for 24 hours. 10 μL varying quantities of SRP (25, 50, 100, 200, 400, 600, 800 μg/mL) and LPS (1 μg/mL) were set as normal group and posititive group, each group was set up with 4 multiple wells, and blank medium group was put in a 37°C, 5 percent CO_2_ cell incubator for a length of time. Then, CCK-8 with 1/10 volume of cell culture media was added to it, and the absorbance of the reaction solution was measured at 450 nm after incubation in a constant temperature incubator.

#### 2.5.3 Assay of phagocytosis

When the confluence of RAW264.7 macrophages reached 80%, the medium was used to dilute the cell density to 1×10^4^ cells/mL. A 96-well plate containing a cell suspension of RAW 264.7 macrophages at a concentration of 100 μL/well was placed in a cell incubator for 24 hours. 100 μL varied doses of SRP (25, 50, 100, 200 μg/mL) and LPS (1 μg/mL) were added and cultured in a cell incubator for 24 hours, adding 100 μL 0.1 percent of the neutral red toμ each well, and the final concentration of neutral red in each well is 1 μg/mL. After washing with PBS, 100 μL cell lysis solution was added to each well and gently shaken for 15 seconds, then left the plate at 37°C for 2 hours. The absorbance of the reaction solution was measured at 540 nm.

#### 2.5.4 Cell scratch experiment

Cell scratch experiment was detected and the method was according to the report of Zubair et al. (17). RAW264.7 macrophages were seeded at a density of 1 ×10^6^ cells per well and cultured in a 6-well plate for 24 hours before being scratched with the tip of a 200 μL pistol to ensure even damage. Then washing with PBS for 3 times to remove the scratched cells, and the medium with different concentrations of SRP (25, 50, 100, 200 μg/mL) and LPS (1 μg/mL) were added and incubated for 24 hours. The medium without drugs was used as the blank control group. The samples were taken and the migration of cells at the same location was recorded.

#### 2.5.5 Extraction of mRNA and RT-PCR

**Table 1** displayed primer sequences that were developed using real-time PCR primer design concepts. Total RNA was isolated using the Trizol procedure, and its concentration and quality were evaluated using a Nanodrop 8000. Then, the obtained RNA were used as templates, cDNA were synthesized by reverse transcription using qPCR (+gDNA) of HiScriptIII RT SuperMix. The fluorescence quantitative reaction system was prepared according to Taq Pro Universal SYBR qPCR Master Mix. After the reaction, the relative gene expression changes were recorded by the 2^-ΔΔCT^ method.

**Table 1:** Sequences for RT-PCR primers.

Primer	Forward (5’-3’)	Reverse (5’-3’)
TNF-α	GACGTGGAACTGGCAGAAGAG	TTGGTGGTTTGTGAGTGTGAG
IL-1β	GGGCCTCAAAGGAAAGAATC	TACCAGTTGGGGAACTCTGC
IL-6	AGTTGCCTTCTTGGGACTG	CAGAATTGCCATTGCACAA
β-actin	ACCCCAGCAAGGACACTGAGCAAG	GGCCCCTCCTGTTATTATGGGGGT

#### 2.5.6 Western blot analysis

Total cell protein was isolated using a protein lysate. Protein was quantified using a kit (Bicinchoninic acid, BCA method), transferred to a PVDF membrane using SDS-PAGE gel electrophoresis, blocked in skim milk for 2 hours, and then incubated at 4°C overnight with a diluted primary antibody. The next day, we washed away the residual primary antibody with TBST solution, added the appropriate secondary antibody, and incubated everything at 26°C for 2 hours. After three washes in TBST buffer solution, any lingering secondary antibodies were removed, and an ECL luminous reagent was poured into an automated chemiluminescence imager. Image J was used to save and examine the data.

### 2.5.7 Statistical analysis

SPSS 21.0 was used to conduct the statistical analysis, and one-way analysis of variance (ANOVA) was employed for the multiple comparisons. In this study, significant differences were defined as those with a probability level of less than 0.05 (represented as “mean ± standard deviation”).

## 3 Results and analysis

### 3.1 Univariate experiments

#### 3.1.1 Effect of ammonium sulfate concentration on SRP yield

**Figure 1:**
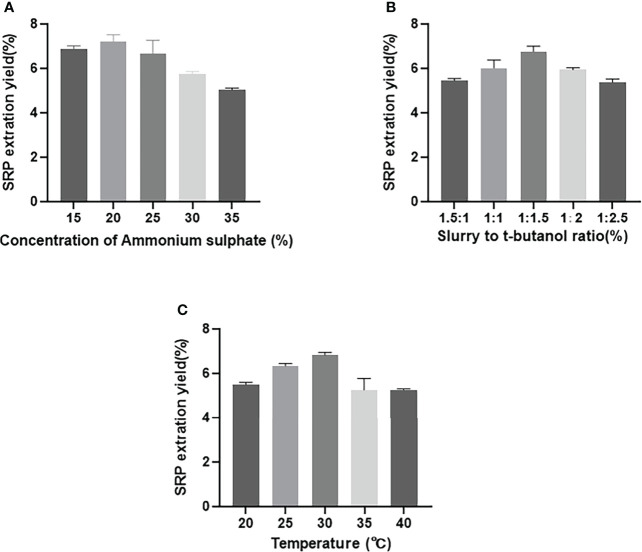
Effect of different factors on SRP yield. **(A)** Concentration of ammonium sulphate; **(B)** Slurry to t-butanol ratio; **(C)** Temperature.

As shown in **Figure 1A**, as the concentration of ammonium sulfate rose from 15%-35%, the yield of SRP increased first and then decreased. When the mass fraction of ammonium sulfate was 20%, the yield reached the maximum value of 7.20%. The extraction yield of polysaccharides was diminished because of the high salt concentration’s potential to disrupt the hydrogen-bond network between SRP and water molecules. Similar to our study, Wang et al. also showed that the extraction efficiency of rice bran polysaccharides decreased with the increase of the concentration of ammonium sulfate (18).

#### 3.1.2 Effect of the sample solution to t-butanol ratio on SRP yield

As shown in **Figure 1B**, the yield of SRP increased gradually with the increasing proportion of t-butanol volume up to a certain point; this was presumably due to increased interaction between increased t-butanol and ammonium sulfate (19). When the ratio of sample solution to t-butanol was 1:1.5 (v/v), the yield reached the maximum value of 7.0%. With further increase of t-butanol, however, the yield of SRP was decreased, thus reducing the overall extraction yield of SRP. This reduction may be due to a lack of sufficient water to hydrate sulfate ions adequately (11).

#### 3.1.3 Effect of the temperature on SRP yield

As shown in **Figure 1C**, the yield of SRP increased gradually with the increase of extraction temperature from 20-30°C; above 30°C, the yield of SRP showed a downward trend. This may be because high temperature accelerated the thermal movement of molecules in the three-phase extraction system, and thus enhanced the hydrophilicity of polysaccharides (20). Therefore, the yield of SRP reached its highest value at the tested temperature, 30°C.

Using the single factor investigation results, an orthogonal test was used to optimize the three extraction parameters of mass fraction of ammonium sulfate, t-butanol volume ratio and extraction temperature, and three levels were set for each factor (**Table 2**). Without considering interaction, an L9(3^3^) orthogonal experiment was conducted to optimize the extraction.

**Table 2:** Orthogonal test factor level table.

Levels	Factors		
	Ammonium sulfate mass fraction (%)	T-butanol volume ratio	Extraction temperature (°C)
1	15	1:1	25
2	20	1:1.5	30
3	25	1:2	35

As can be seen from the R-value in the **Table 3**, the influence of these three parameters on the yield of polysaccharides is as follows: B (t-butanol volume ratio) > C (extraction temperature) > A (ammonium sulfate mass fraction). On this basis, the optimum extraction conditions were determined as follows: mass fraction of ammonium sulfate, 20%; volume ratio of sample solution to t-butanol, 1:1.5; extraction temperature, 35°C.

**Table 3:** Results and analysis of orthogonal experiment of SRP.

No	A	B	C	Yield (%)
1	1	1	1	5.72
2	1	2	2	6.56
3	1	3	3	5.63
4	2	1	2	6.13
5	2	2	3	7.12
6	2	3	1	5.81
7	3	1	3	6.5
8	3	2	1	6.06
9	3	3	2	6.27
K1	17.91	18.35	17.59	
K2	19.06	19.74	18.96	
K3	18.83	17.71	19.25	
R	1.15	2.03	1.66	

### 3.2 Physicochemical properties

#### 3.2.1 Physicochemical composition and monosaccharide composition

As shown in **Table 4**, the extraction yield of SRP was 6.85%, and the carbohydrate content of SRP was 56.18%. The protein content was 6.78%, and the polyphenol content was 1.73%. The XRD pattern showed a small diffraction peak at 20°, which indicated that SRP has an amorphous structure. Its crystallinity was 18.21%. The crystal structure is directly determined by properties such as elasticity and swelling (21).

**Table 4:** Physicochemical composition and molecular weight.

Sample	SRP
Yield (%)	6.85 ± 0.13
Carbohydrate (%)	56.78 ± 1.34
Protein (%)	6.78 ± 0.15
Polyphenol (%)	1.73 ± 0.0050
Degree of crystallinity (%)	18.21 ± 0.34
Molecular weight (kDa)	
Weight-average molecular weight (Mw)	27.52
Number-average molecular weight (Mn)	26.07
Polymer dispersity index (PDI)	1.06

Molecular weight is an essential physicochemical parameter closely related to the biological activity of polysaccharides. The weight average molecular weight (Mw) of SRP was 27.52 kDa, and its number average molecular weight (Mn) of SRP was 26.07 kDa, which was similar to Chen (22). The polymer dispersion index Mw/Mn of SRP was 1.06, and the closer the dispersion index was to 1, the more evenly the polysaccharide was distributed.

#### 3.2.2 Monosaccharide composition of SRP

Monosaccharide composition results (**Table 5**) showed that the SRP sample was composed of fucose, rhamnose, arabinose, galactose, glucose, xylose, fructose and glucuronic acid in a molar ratio of 4.19: 3.46: 3.26: 26.80: 35.79: 7.92: 8.65: 9.92. Galactose and glucose were the most abundant. The results showed that SRP was heteropolysaccharide. Liu et al. (2) identified mannose, galactose and glucose as the main monosaccharide components in SRP. Similarly, Diego Morales et al. found that the monosaccharide components of *Lentinula edodes* polysaccharides are mainly glucose, galactose and mannose (23). Different sources of raw materials and preparation conditions may be responsible for the differences.

**Table 5:** Monosaccharide composition of SRP.

Monosaccharides	Molar ratio (%)
Fucose	4.19
Arabinose	3.46
Rhamnose	3.26
Galactose	26.8
Glucose	35.79
Xylose	7.92
Fructose	8.65
Glucuronic acid	9.92

#### 3.2.3 SEM analysis

**Figure 2:**
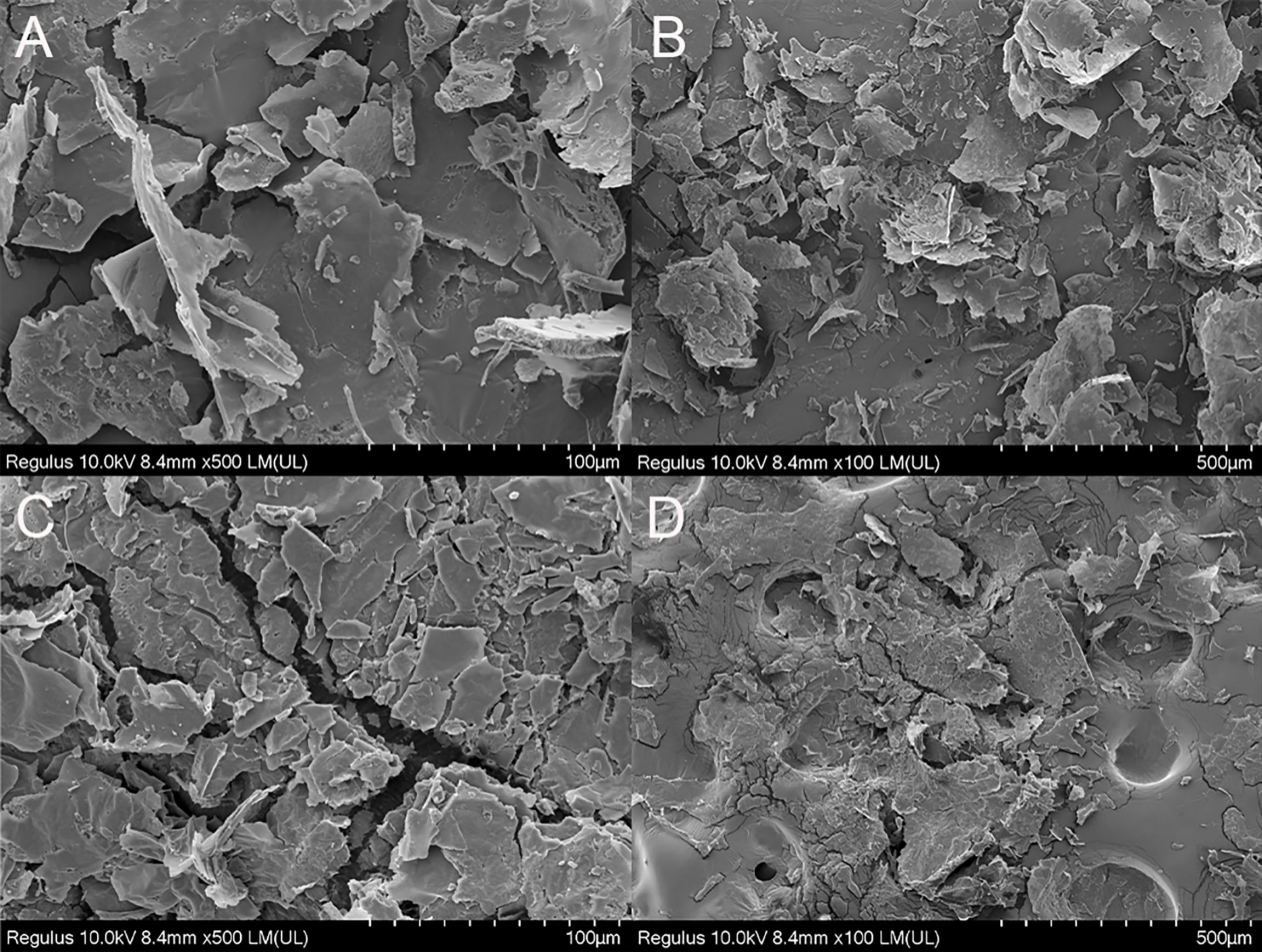
SEM images (magnification 100× and 500×). **(A, C)** (100 µm); **(B, D)** (500 µm).

The morphology of SRP was characterized by SEM. SRP had a rough surface, irregular flake and massive distribution, and pores on the surface after magnification (**Figure 2**). These microstructure characteristics may be caused by the destruction of cell wall structure and the decomposition of polysaccharide aggregates during the three-phase extraction processes. The surface topographic characteristics and microstructure change of SRP may be related to its physicochemical properties and antioxidant activity (24). SRP obtained by the three-phase extraction method is due to the joint action of ammonium sulfate and tert-butanol, which is affected by various forces and forms an obvious flake structure.

### 3.3 Antioxidant activity analysis *in vitro* of SRP

#### 3.3.1 DPPH radical scavenging ability of SRP

**Figure 3:**
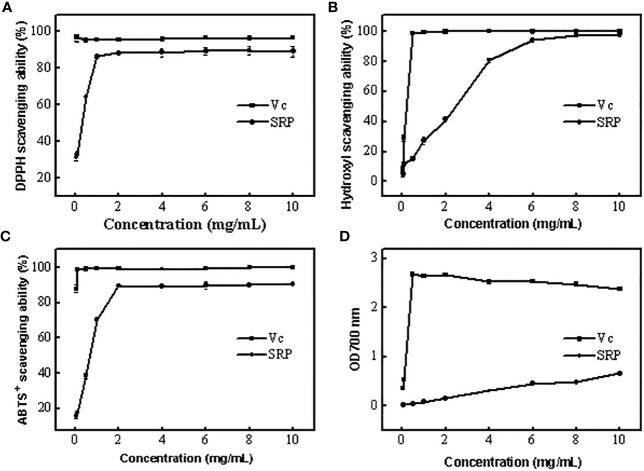
Antioxidant activity of SRP **(A-D)** DPPH, Hydroxyl, ABTS^+^ radical scavenging ability and reducing power.

The antioxidant potential of polysaccharides may be easily guaranteed by measuring their capacity to scavenge DPPH free radicals. In general, the eliminated ability of polysaccharides to DPPH free radicals relies on the hydrogen supply ability of the antioxidant (25). **Figure 3A** displayed SRP’s capacity to scavenge DPPH free radicals. The scavenging activities of SRP reached 88.60 percent when the SRP reached 2 mg/mL. The result indicated that SRP had the ability to clear DPPH, which was similar to Wang et al. (26).

#### 3.3.2 Hydroxyl radical scavenging ability of SRP

Excessive levels of hydroxyl radical, one of the most powerful free radicals in the body, may disrupt the delicate equilibrium of the body. Upon contact with nearby biomolecules, hydroxyl radicals may cause extensive damage, and in extreme cases, even cell death (27). **Figure 3B** displayed SRP’s ability to quench hydroxyl radicals. Generally, SRP had potential scavenging activity on hydroxyl radical. SRP’s scavenging activity rose to 94.46 percent at 6 mg/mL. When the SRP concentration was 10 mg/mL, its scavenging efficiency was 98.06%. SRP was shown to have a clear capacity to scavenge hydroxyl radicals, as shown by the findings. SRP had the potential to remove hydroxyl radical because it has a better scavenging power on hydroxyl radical.

#### 3.3.3 ABTS^+^ radical scavenging ability of SRP

The antioxidant potential of polysaccharides may be easily neutralized using the ABTS^+^ free radical technique (28). ABTS^+^ free radical scavenging experiments are based on electron transfer from antioxidants to ABTS^+^ free radicals. SRP’s capacity to scavenge ABTS^+^ radicals were shown in **Figure 3C**. As a positive control, the scavenging power of Vc remained stable between 88.13% and 99.83% in the range of experimental concentrations. At 2 mg/mL, SRP showed an 89.44 percent increase in scavenging activities. These results showed that SRP had the ability to scavenge ABTS^+^ radical.

#### 3.3.4 Reducing power of SRP

In **Figure 3D**, the lowering power of SRP compared to the positive control, Vc. The concentration of SRP has a positive correlation with the reducing power. SRP had an absorbance of 0.66 at 700 nm at a dosage of 10 mg/mL. These findings demonstrated that SRP has some degree of astringent activity.

### 3.4 Measurement of immunomodulatory activities of SRP

#### 3.4.1 Cell viability

**Figure 4:**
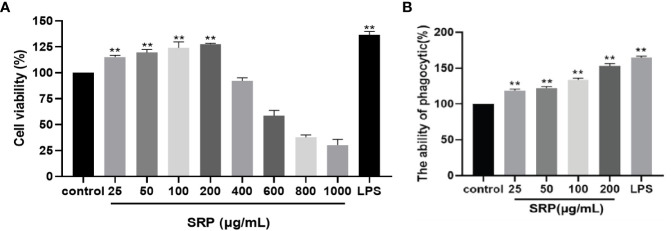
Cell viability and phagocytic capacity of the SRP. **(A)** Cell viability; **(B)** Phagocytic capacity. Significant differences with the control group: *p<0.05 and **p<0.01.

The results of testing the viability of cells treated with SRP were shown in **Figure 4A**. By comparing with the blank group, different concentrations of SRP had different effects on RAW264.7 macrophages activity. When SRP ≤ 200 µg/mL, SRP can significantly promote cell growth (*p*<0.05) indicating that SRP had no toxicity to cells. Concentrations greater than or equal to 400 µg/mL inhibited cell proliferation, indicating some toxicity.

Therefore, based on these results, a concentration between 25-200 µg/mL could be used for experimental treatment of cells, specifically for evaluating the immune regulatory effect of SRP on RAW264.7 macrophages.

#### 3.4.2 Effect of SRP on phagocytosis of RAW264.7 macrophages

The effect of SRP on phagocytosis was detected by macrophage uptake of neutral red (29). The results were shown in **Figure 4B**. When the concentration of SRP was between 25-200 μg/mL, all samples significantly promoted the uptake of neutral red by RAW264.7 macrophages compared with the blank control group (*p*<0.05). The results showed that SRP significantly promoted the uptake of neutral red by macrophages, thus further regulating immune activity.

#### 3.4.3 Effect of SRP on RAW264.7 macrophages migration

**Figure 5:**
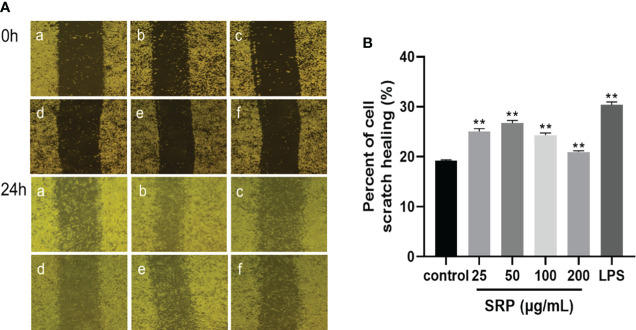
Results of the macrophages RAW264.7 scratch experiment. **(A)** Image of the cell scratch: a-f indicates the control, LPS, SRP (25, 50, 100, 200 μg/mL) groups; **(B)** Percent of cell scratch healing. Significant differences with the control group: *p< 0.05 and **p< 0.01.

The most direct method to detect cell migration is the scratch test (30). As shown in **Figure 5**, RAW264.7 macrophages were treated with LPS (1 μg/mL) and different concentrations of SRP (25-200 μg/mL), and the percent of cell scratch healing on the macrophages was observed. The results showed that SRP significantly improved cell scratch healing at all tested concentrations between 25-200 μg/mL compared with the blank group (*p*<0.05), and the scratch healing rate of the RAW264.7 macrophages at 25-50 μg/mL SRP was higher than that at 100-200 μg/mL.

#### 3.4.4 RT-PCR test results

**Figure 6:**
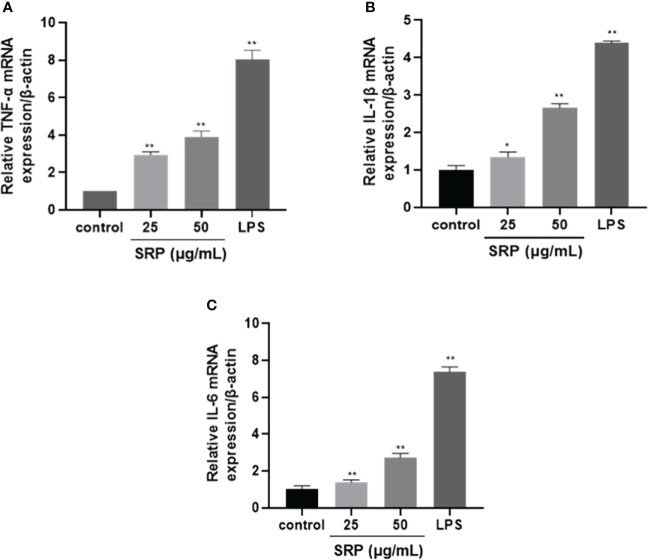
Effects of SRP on the expression of cytokine genes in RAW264.7 macrophages. **(A-C)** Expression of TNF-α, IL-1β and IL-6 mRNA. Significant differences with the control group: *p< 0.05 and **p< 0.01.

Cytokines are produced by activated immune cells and are considered to be major immune mediators (31). The transcription levels of the three cytokine genes IL-1β, IL-6 and TNF-α were detected by RT-PCR. **Figure 6** showed that transcription levels of all three were significantly increased within the limits of SRP (25-50 μg/mL) or LPS (1 μg/mL) groups compared with the blank group (*p*<0.05). The result was similar to the results reported by Liu on the immunomodulatory activity of *Sinonovacula constricta* polysaccharide (32).

#### 3.4.5 Protein expression of TLR4/NF-κB pathway

NF-κB plays a crucial role in immune regulation and inflammatory responses (33). TLR4 is a significant member of the Toll-like receptor family, and breakthroughs have been made in research on TLR4. A variety of signaling pathways have been confirmed to be related to mediating immune responses (34). To determine whether the immune activity of SRP is mediated by the TLR4/NF-κB signaling pathway, we investigated western blot analysis was performed to analyze the effect of SRP on the expression levels of key proteins in the TLR4/ NF-κB signaling pathway in RAW 264.7 cells.

**Figure 7:**
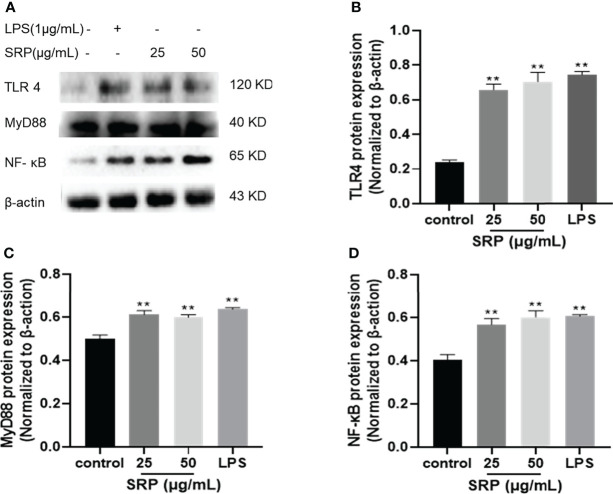
Effect of SRP on the protein expression levels of the TLR4/NF-κB pathway. **(A)** Western blot analysis of protein bands; **(B)** TLR4; **(C)** MyD88; **(D)** NF-κB protein expression. Significant differences with the control group: *p< 0.05 and **p< 0.01.

**Figure 7** showed the influence of SRP on the expression levels of the key proteins TLR4, MyD88 and NF-κB in the TLR4/NF-κB signaling pathway (β-actin as internal reference). Compared with the positive control group, SRP groups showed decreased the protein expression levels. Compared with the blank group, SRP (25-50 μg/mL) and LPS groups showed significantly increased expression of TLR4, MyD88 and NF-κB protein (*p*<0.05). We speculated that SRP activates RAW264.7 macrophages by up-regulating the expression of key proteins in the TLR4/NF-κB pathway.

#### 3.4.6 Expression of autophagy proteins

Autophagy is a cellular mechanism of self-degradation, transformation and energy production. Autophagy is closely related to inflammation. The activation of pattern recognition Toll-like receptor can induce autophagy, and autophagy can regulate the inflammatory response. Defective autophagy can induce inflammation (35). LC3 is a hallmark protein of autophagy. p62 is a substrate for autophagy degradation; it plays an important role in the recognition and encapsulation of degraded substrates. Beclin 1 over expression promotes autophagy in mammalian cells, and the expression increases with the enhancement of autophagy. LC3, Beclin 1 and p62 are all used as indicators of autophagy (36).

**Figure 8:**
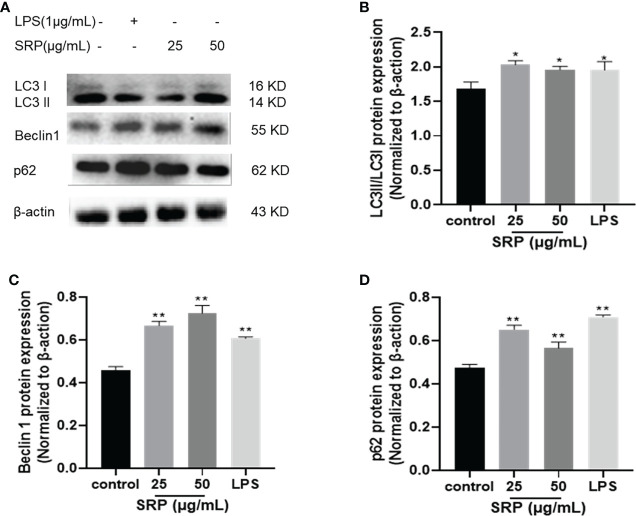
Expression of autophagic proteins. **(A)** Western blot analysis of protein bands; **(B-D)** LC3, Beclin1 and p62 protein expression. Significant differences with the control group: *p< 0.05 and **p< 0.01.

Autophagic protein expression results were shown in **Figure 8**. The expression of autophagic protein in the positive control group was higher than in the blank group. Compared with the positive control group, the protein expression levels of LC3 and Beclin 1 increased and the protein expression levels of p62 decreased in SRP with different mass concentrations. Compared with the blank group, the protein expression levels of LC3, Beclin 1 and p62 in different mass concentrations of SRP were significantly increased (*p*<0.05), which indicated that SRP can promote autophagy in cells.

## 4 Discussion

Recently, many studies have been carried out on the extraction of polysaccharides from *S. rugosoannulata*, which due to its better biological. The main extraction methods are solvent extraction, ultrasonic extraction, alkali-assisted extraction (2), etc. However, these extraction methods are complex, typically requiring a long processing time.

As a new protein separation and purification technology, three-phase extraction is a safe, effective, and environmentally friendly green technology, including salting out, isoelectric point precipitation and solvent precipitation (37). However, most researchers mainly focus on the protein phase (38). It is rarely reported that polysaccharides are isolated and purified from edible fungi by three-phase extraction. Ours may be the first attempt to isolate SRP by three-phase extraction. Three-phase extraction is relatively simple, safe, and environmentally friendly compared to conventional extraction methods. Furthermore, the method preserves the biological activity of the polysaccharides better than the existing method.

In this study, single factor and multiple factor orthogonal tests were used to obtain the optimal extraction conditions for extracting polysaccharides. These conditions were: mass fraction of ammonium sulfate, 20%; volume ratio of sample solution to t-butanol, 1:1.5; and extraction temperature, 35°C. Under these conditions, the yield of SRP was 6.85% ± 0.13%, which was higher than that of the traditional method of water extraction and alcohol precipitation (39).

The physiological functions of polysaccharides are closely related to structure. Some scholars have studied the structure and function of polysaccharides of *S. rugosoannulata*. Chen et al. (22) obtained a polysaccharide by hot water extraction, and proved this structure was mainly composed of five monosaccharides, predominaatly xylose, galactose and glucose, with a relative molecular weight of about 22 kDa. Liu et al. (2) isolated two components, SRP-1 and SRP-2 from the fruiting body of *S. rugosoannulata*. Both components contained glucose and galactose, but SRP-2 also contained ribose and uronic acid. We speculate that these differences in monosaccharide compositions of *S. rugosoannulata* were due to differences in the source material.

In our study, the polysaccharide from *S. rugosoannulata* was extracted by three-phase extraction. According to our analysis, the polysaccharide was heteropolysaccharide composed of 8 monosaccharides, and the molecular weight was 27.52 kDa, which was larger than that polysaccharides obtained by hot water extraction (22). The molecular mass of SRP was bigger, the monosaccharide composition was richer, and its physiological activity was stronger. At the same time, this method also had a positive effect on the structure modification, which can interrupt the long molecular chain of polysaccharides and the change of molecular aggregation state, so that the structure of polysaccharides can be changed. Finally, three-phase extraction affects the biological activity and chemical and physical properties, such as improving the antioxidant activity of the polysaccharides (40).

Polysaccharides can improve the immune activity of cells *in vitro*. MOP-3, a novel polysaccharide extracted from *Moringa oleifera* leaves, can enhance the ability of macrophages to secrete reactive oxygen species (ROS), nitric oxide (NO), interleukin-6 (IL-6) and tumor necrosis factor α (TNF-α), indicating that the polysaccharide can activate macrophages to produce cytokines, thus achieving the purpose of fighting pathogens (41). In this experiment, compared with blank control group, the the SRP-treated group showed increased proliferation, phagocytosis and cell migration of 25-200 μg/mL.

Numerous studies have shown that polysaccharides can activate intracellular signaling pathways through TLR4 receptor-mediated macrophages, promote the release of related cytokines, and play immunomodulatory roles. For example, mushroom polysaccharides can activate peritoneal macrophages through the TLR4/NF-κB pathway, and significantly enhance the secretion of cytokines (42). Our study results showed that SRP significantly enhanced the protein expression of TLR4, MyD88 and NF-κB in the range of 25-50 μg/mL, and activated the TLR4-mediated MyD88 dependent pathway, which confirmed that TLR4/NF-κB signaling pathway is one of the important pathways through which polysaccharides exert immunomodulatory effects.

Autophagy is a highly conserved metabolic pathway in organisms. It can selectively degrade intracellular harmful components and play an important role in regulating immunity (35). At present, no articles related to SRP and autophagy have been found. So, this study confirmed that SRP can up-regulate autophagy related proteins LC3 and Beclin1, and down-regulate the expression of p62 protein to induce increased autophagy. This indicates that the mechanism of SRP’s effect on inflammation is related to autophagy induction. Moreover, the expression levels of related proteins and autophagy proteins in the TLR4/NF-κB signaling pathway were significantly increased in the macrophage SRP groups treated with 25-50 μg/mL, suggesting that SRP can activate the TLR4/NF-κB signaling pathway and autophagy.

## 5 Conclusion

In this study, a polysaccharide was extracted from the mushroom *Stropharia rgosoanmulata*, using a new method, three-phase extraction. Extraction parameters were optimized as follows; mass fraction of ammonium sulfate, 20%; volume ratio of sample solution to t-butanol, 1:1.5; and the extraction temperature, 35°C. With these parameters, the yield of polysaccharides was 6.85% ± 0.13%. SRP had the molecular weight of 27.52 kDa. SEM showed that SRP surface was rough and flakey. XRD analysis showed that it had both crystalline and amorphous structures. The results of antioxidation experiments showed that SRP has antioxidant activity. The results of CCK-8 method showed that the polysaccharide concentration in the range of 25-200 μg/mL promoted cell proliferation and had no cytotoxicity. This means that SRP is safe for clinical applications. RT-PCR results confirmed that SRP can significantly promote the release of cytokines such as IL-6 and TNF-α from peritoneal macrophages in the range of 25-100 μg/mL, and the purpose of immune enhancement by enhancing the activity of cytokines. Western blot results showed that SRP induced macrophages RAW264.7 to activate the TLR4/NF-κB signaling pathway of autophagy.

In conclusion, *S. rugosoannulata* has antioxidant and immunomodulatory activities. It appears to be safe. Thus, *S. rugosoannulata* has a broad prospect for further development as a new immunomodulator and antioxidant..

## Data availability statement

The datasets presented in this study can be found in online repositories. The names of the repository/repositories and accession number(s) can be found in the article/**Supplementary Material**.

## Author contributions

XL, ZZ: Research concept, Methodology, Data extraction, Analysis, Draft writing. XM: Resource searching, Verification, Formal analysis, Supervision, Manuscript reviewing and editing. YW: Funding sponsorship. LW, HZ, YJ: Resources, Methodology, Project administration, Supervision, Manuscript reviewing and editing. BM, JL: Resource searching, Manuscript reviewing and editing. All authors contributed to the article and approved the submitted version.
