# Supplementary figures and images for: Three-phase extraction of polysaccharide from Stropharia rugosoannulata: Process optimization, structural characterization and bioactivities
Source: Front Immunol. 2023 Jan 11;13:994706. doi: 10.3389/fimmu.2022.994706 (PMC9878848; doi:10.3389/fimmu.2022.994706)

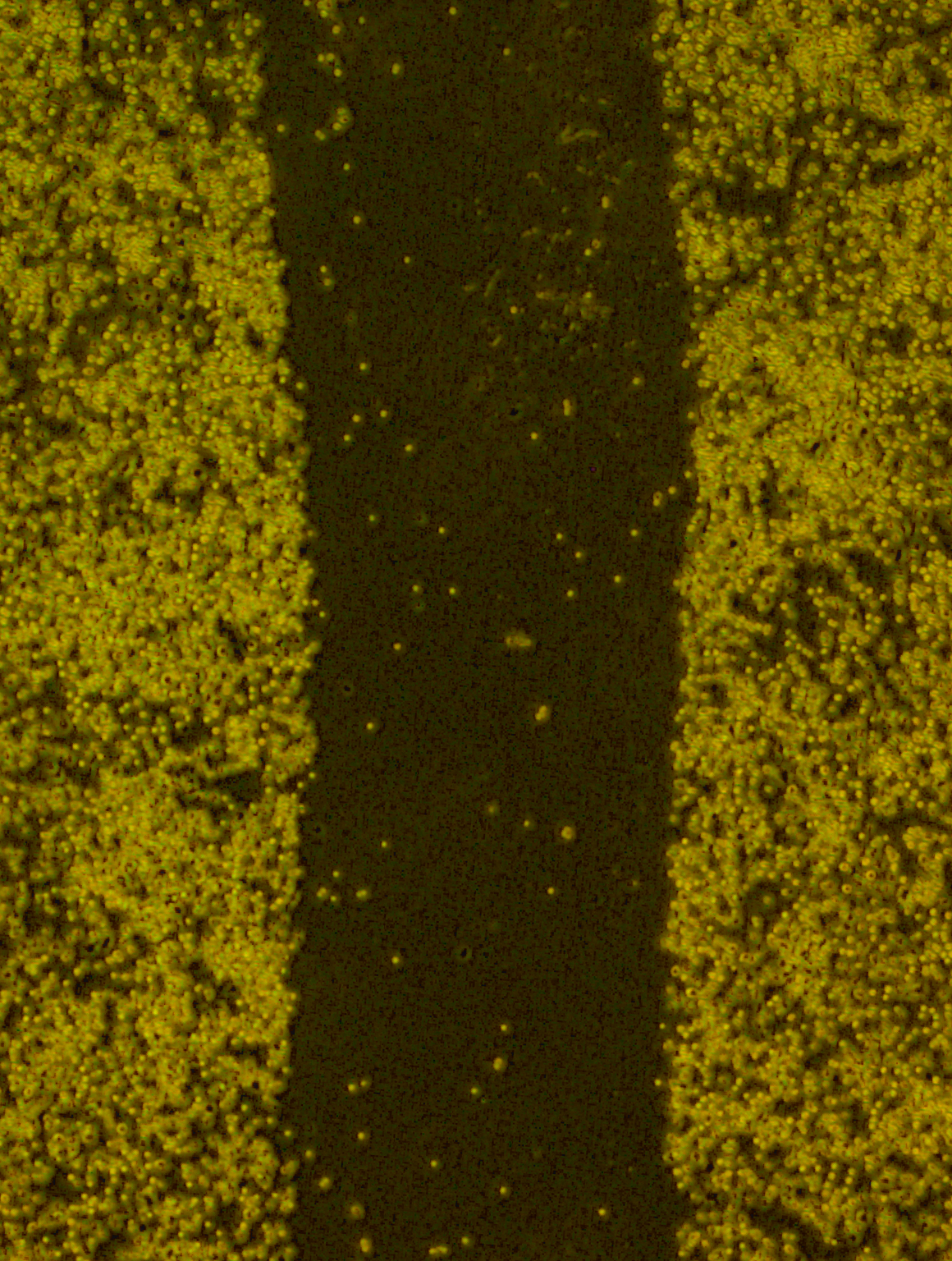

Supplement: Supplementary file 1 [file DataSheet_1.zip › Cell scratch experiment/100 0h.png]

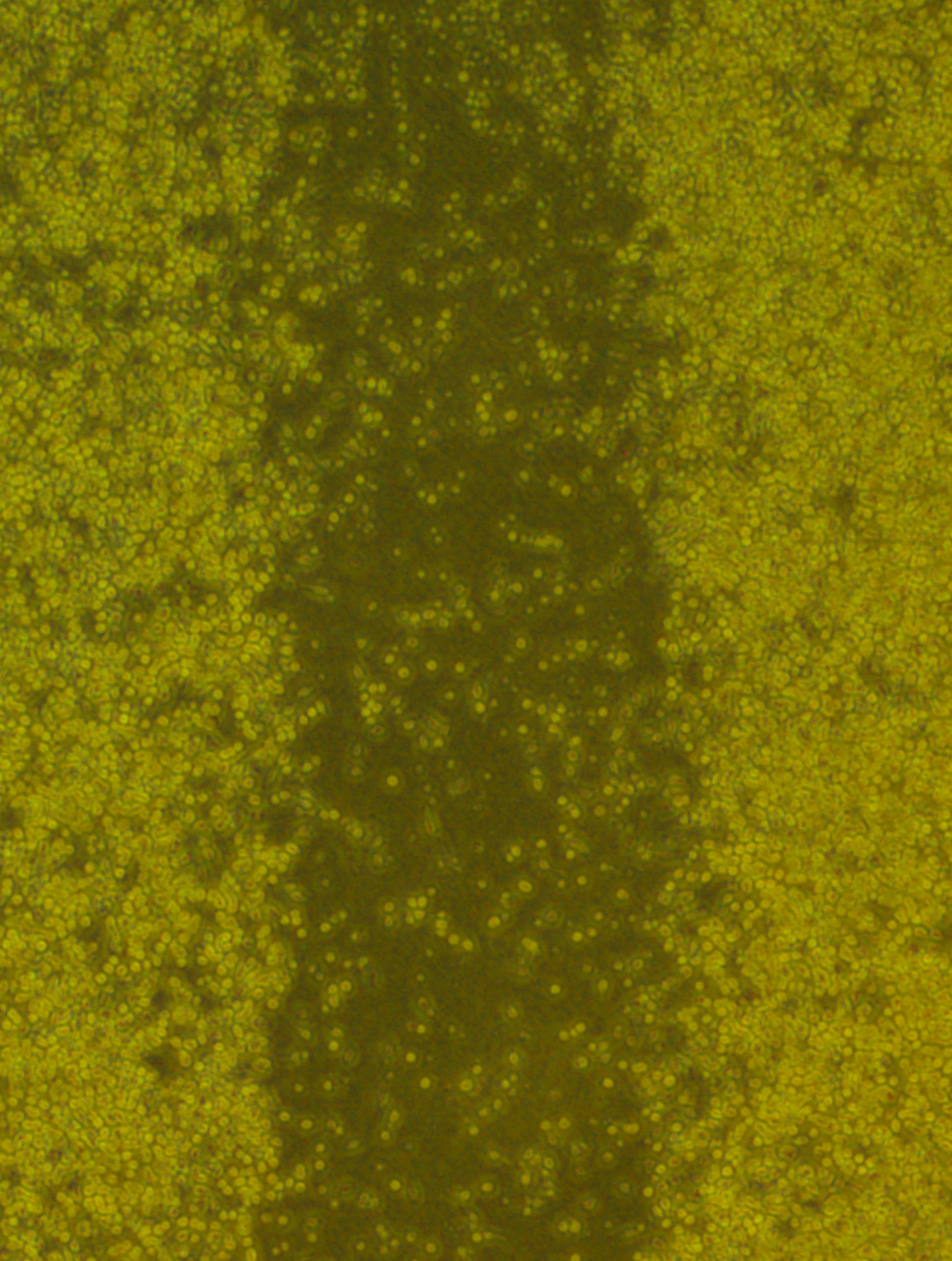

Supplement: Supplementary file 1 [file DataSheet_1.zip › Cell scratch experiment/100 24h.png]

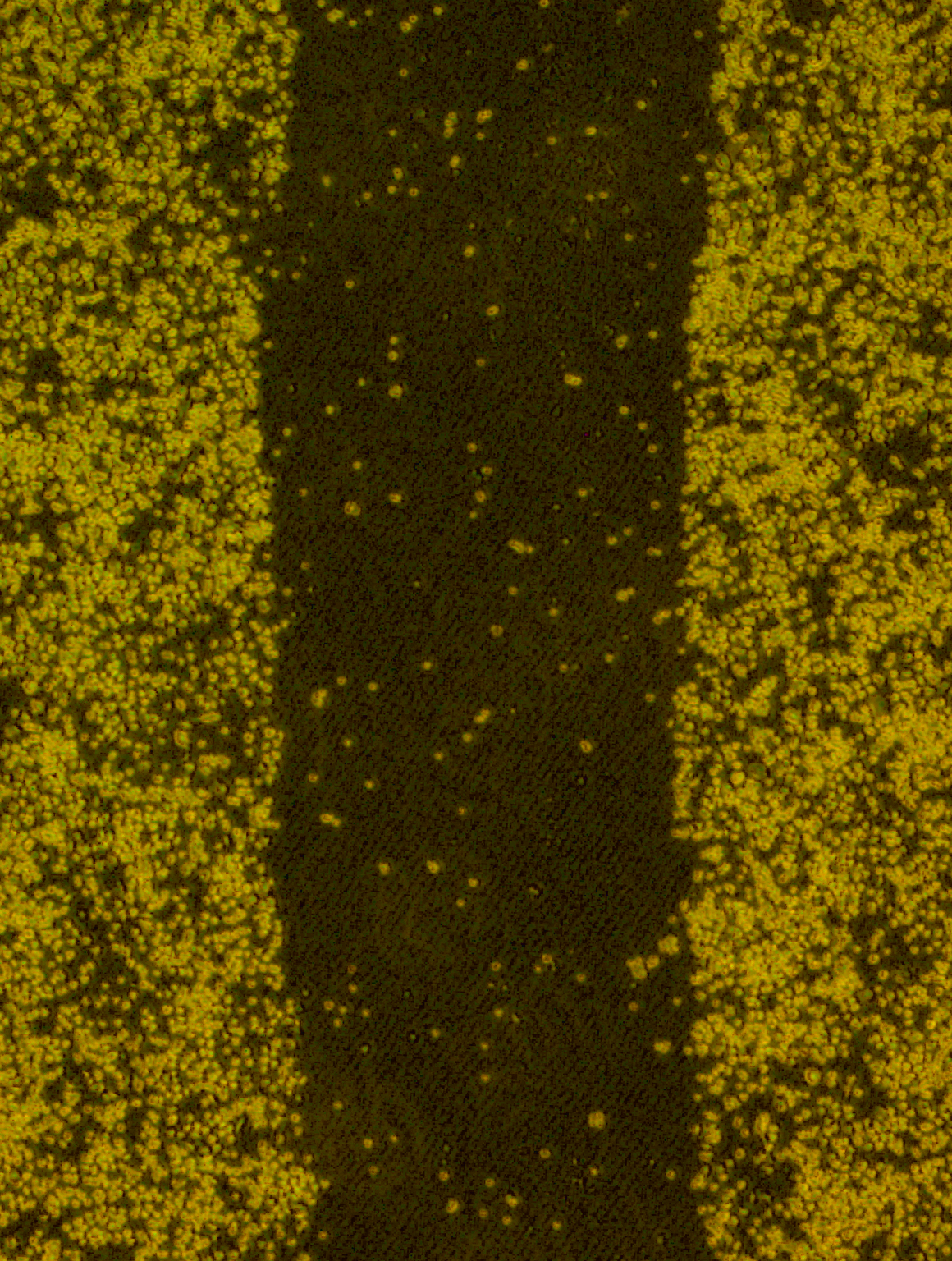

Supplement: Supplementary file 1 [file DataSheet_1.zip › Cell scratch experiment/200 0h.png]

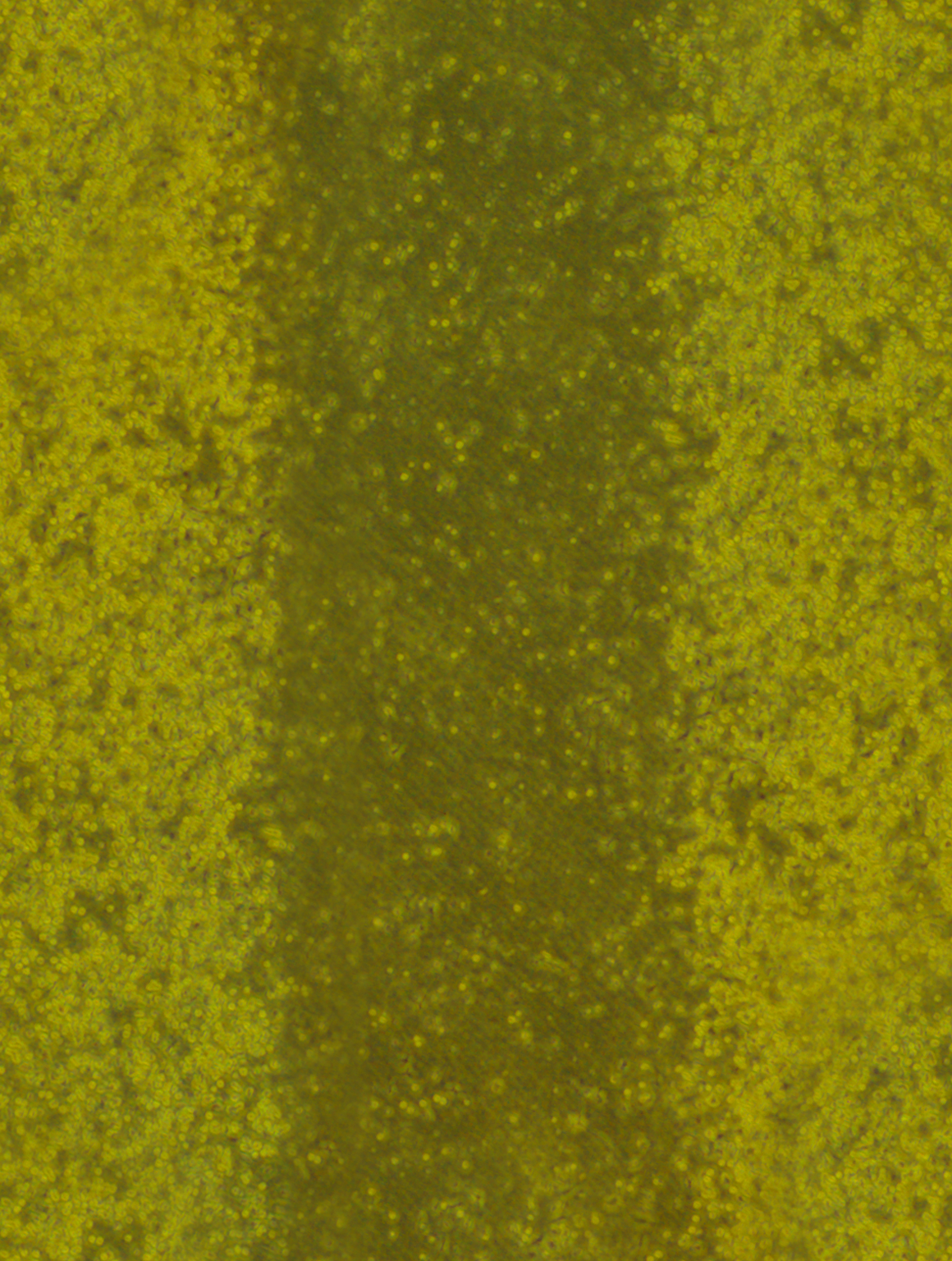

Supplement: Supplementary file 1 [file DataSheet_1.zip › Cell scratch experiment/200 24h.png]

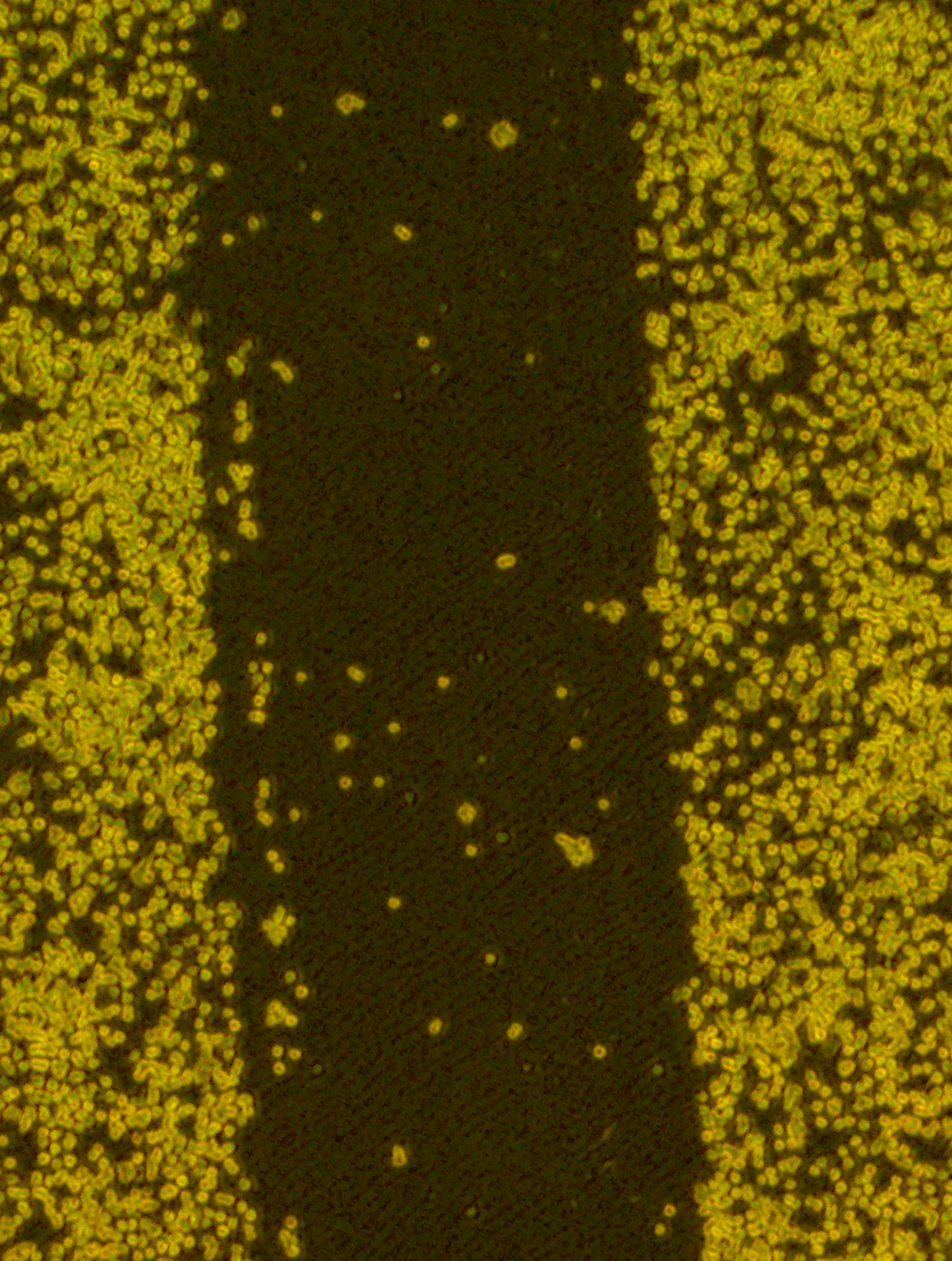

Supplement: Supplementary file 1 [file DataSheet_1.zip › Cell scratch experiment/25 0h.png]

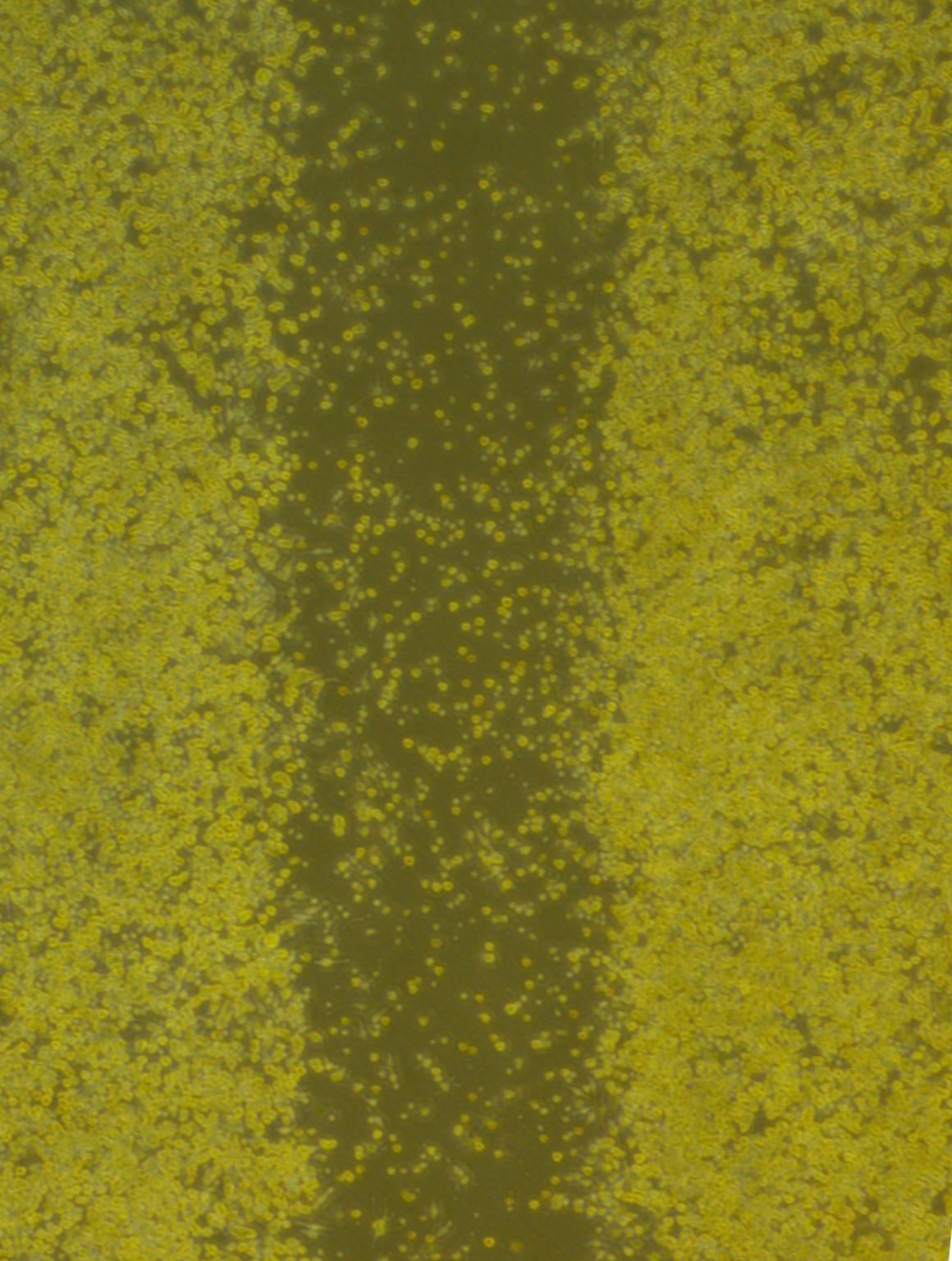

Supplement: Supplementary file 1 [file DataSheet_1.zip › Cell scratch experiment/25 24h.png]

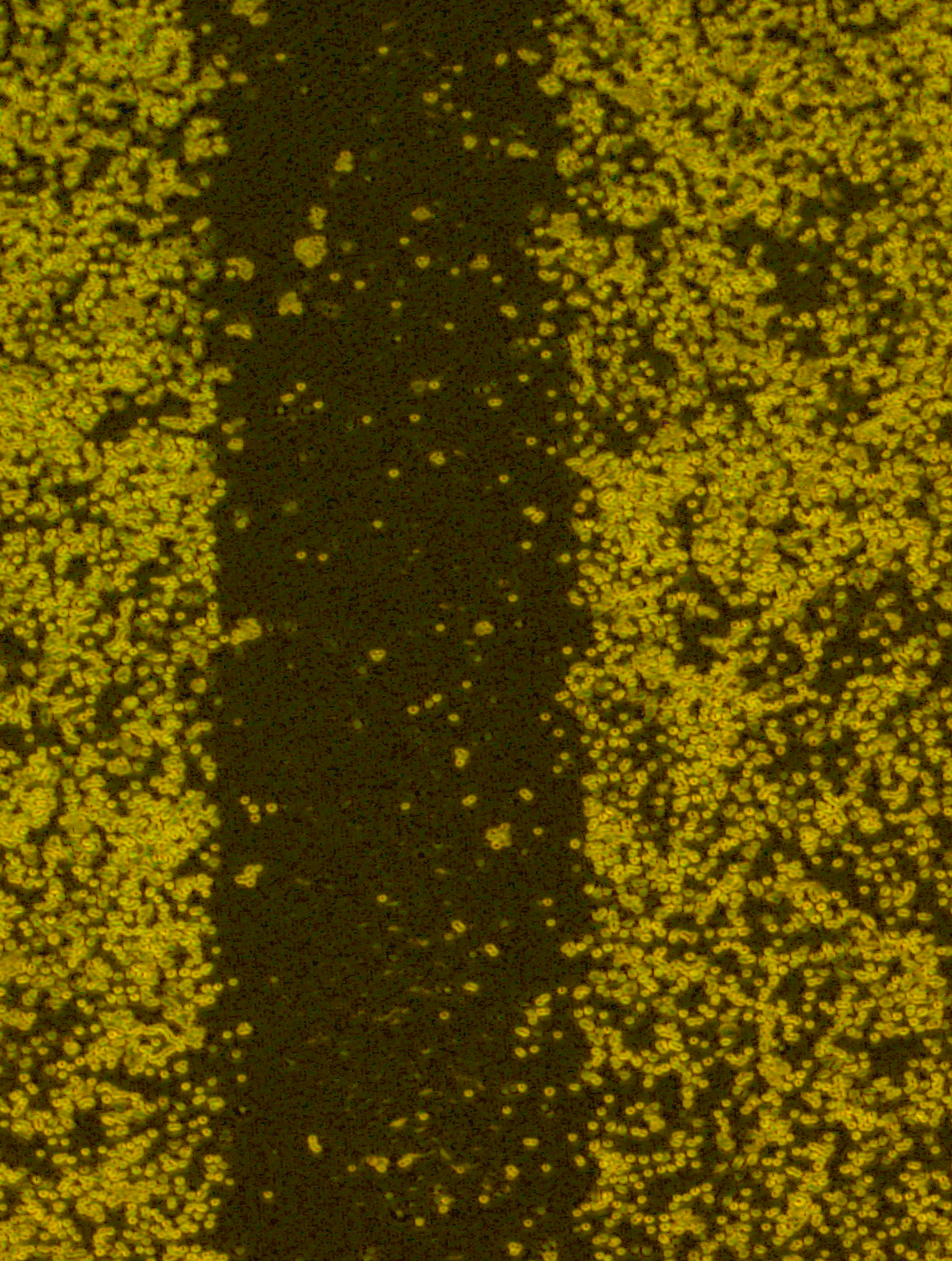

Supplement: Supplementary file 1 [file DataSheet_1.zip › Cell scratch experiment/50 0h.png]

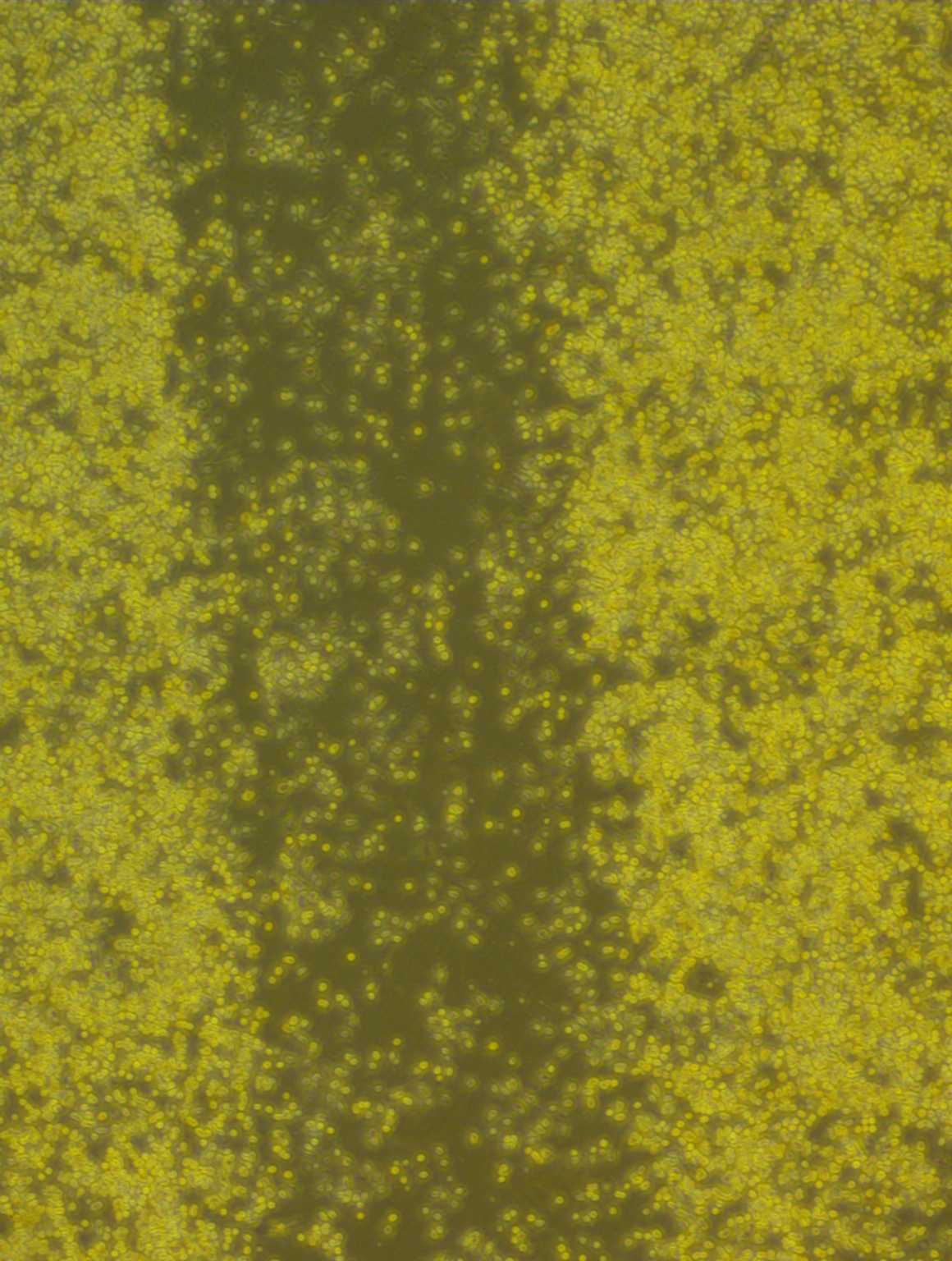

Supplement: Supplementary file 1 [file DataSheet_1.zip › Cell scratch experiment/50 24h.jpg]

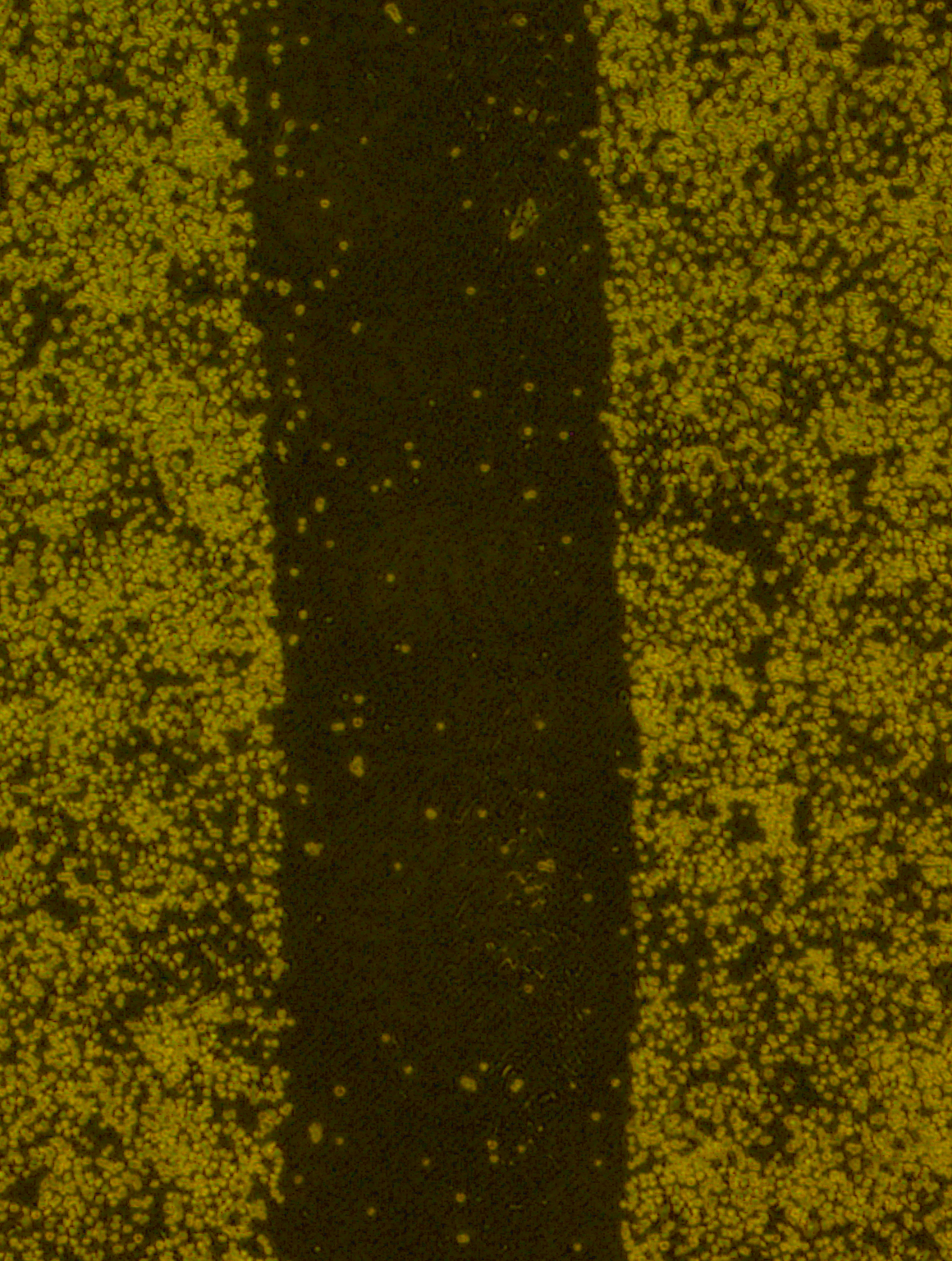

Supplement: Supplementary file 1 [file DataSheet_1.zip › Cell scratch experiment/LPS 0h.png]

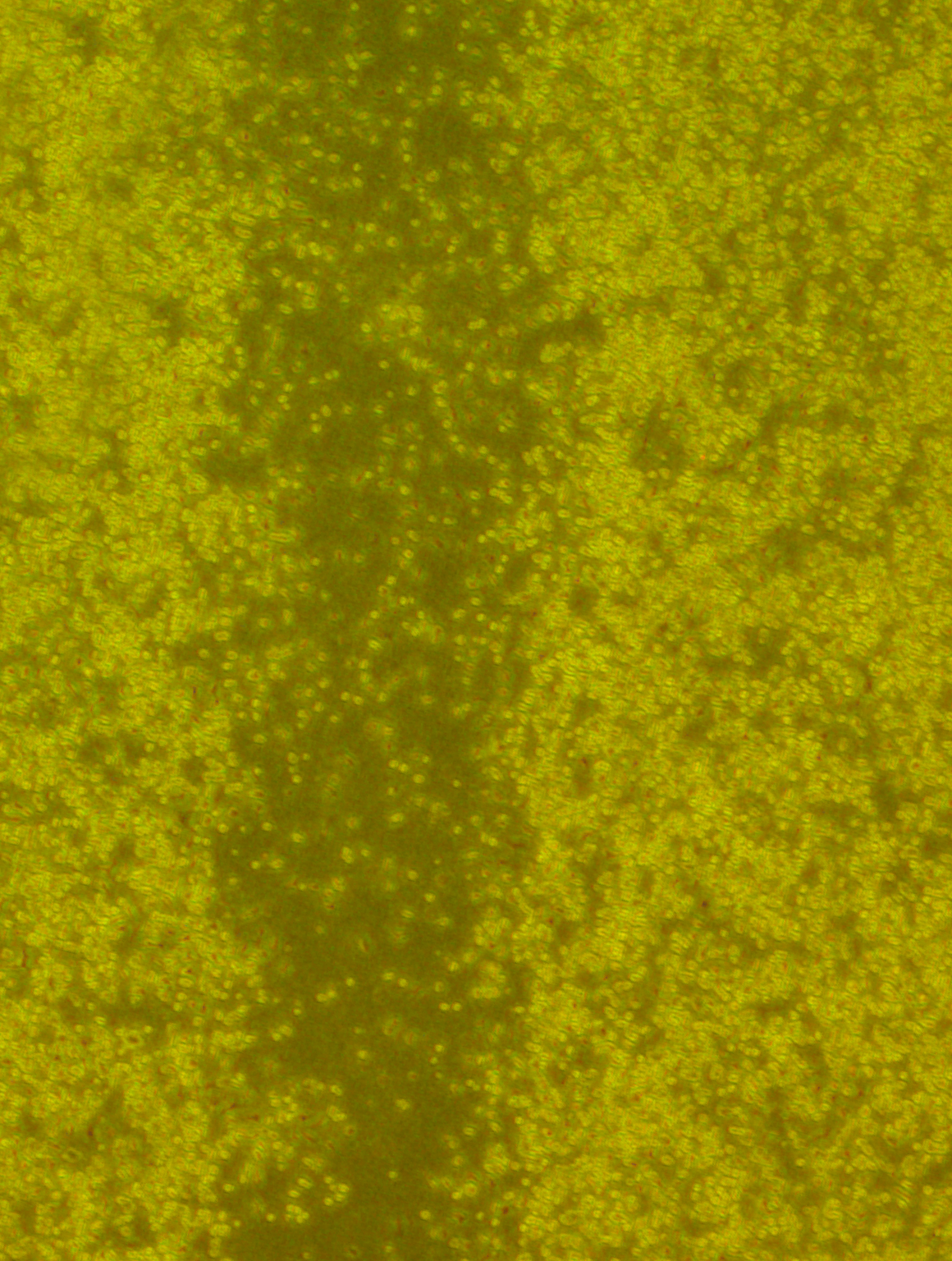

Supplement: Supplementary file 1 [file DataSheet_1.zip › Cell scratch experiment/LPS 24h.png]

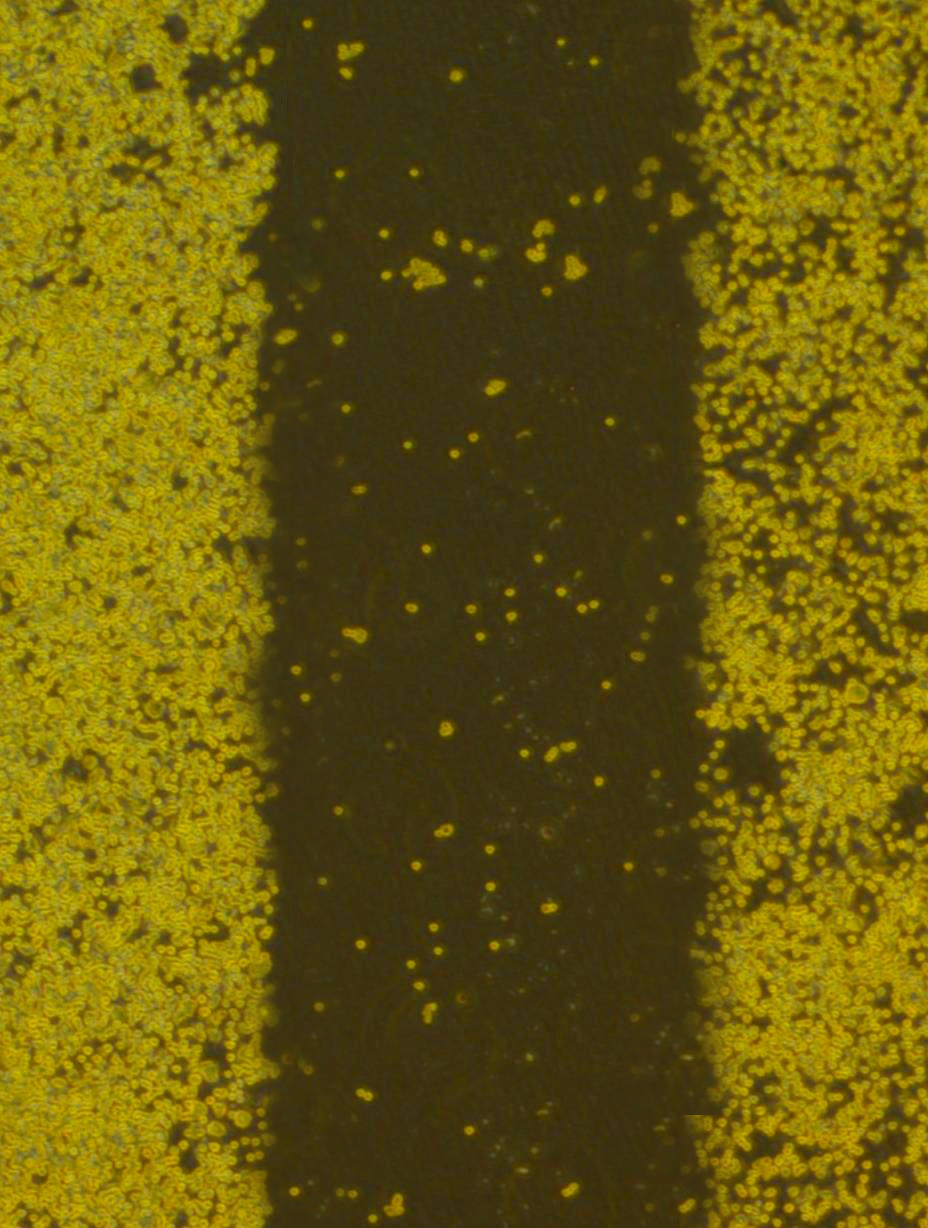

Supplement: Supplementary file 1 [file DataSheet_1.zip › Cell scratch experiment/┐╒ 0h.png]

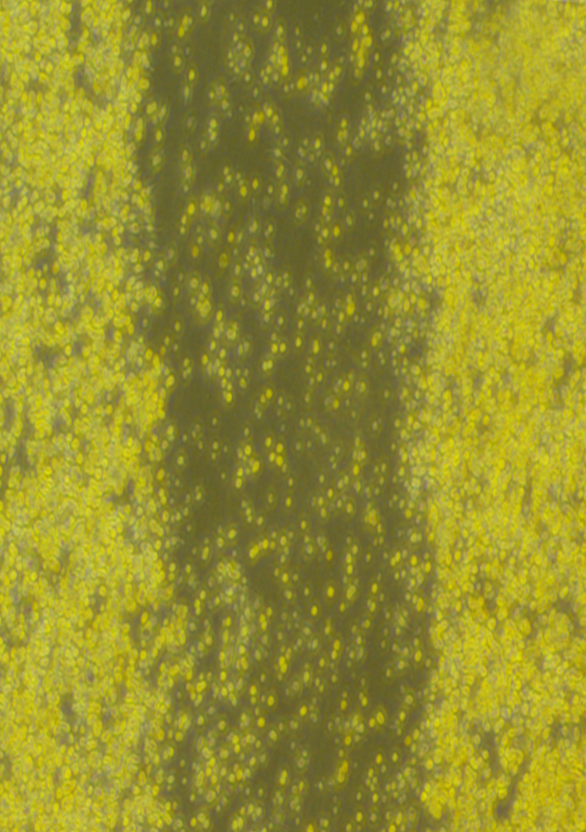

Supplement: Supplementary file 1 [file DataSheet_1.zip › Cell scratch experiment/┐╒ 24h.png]

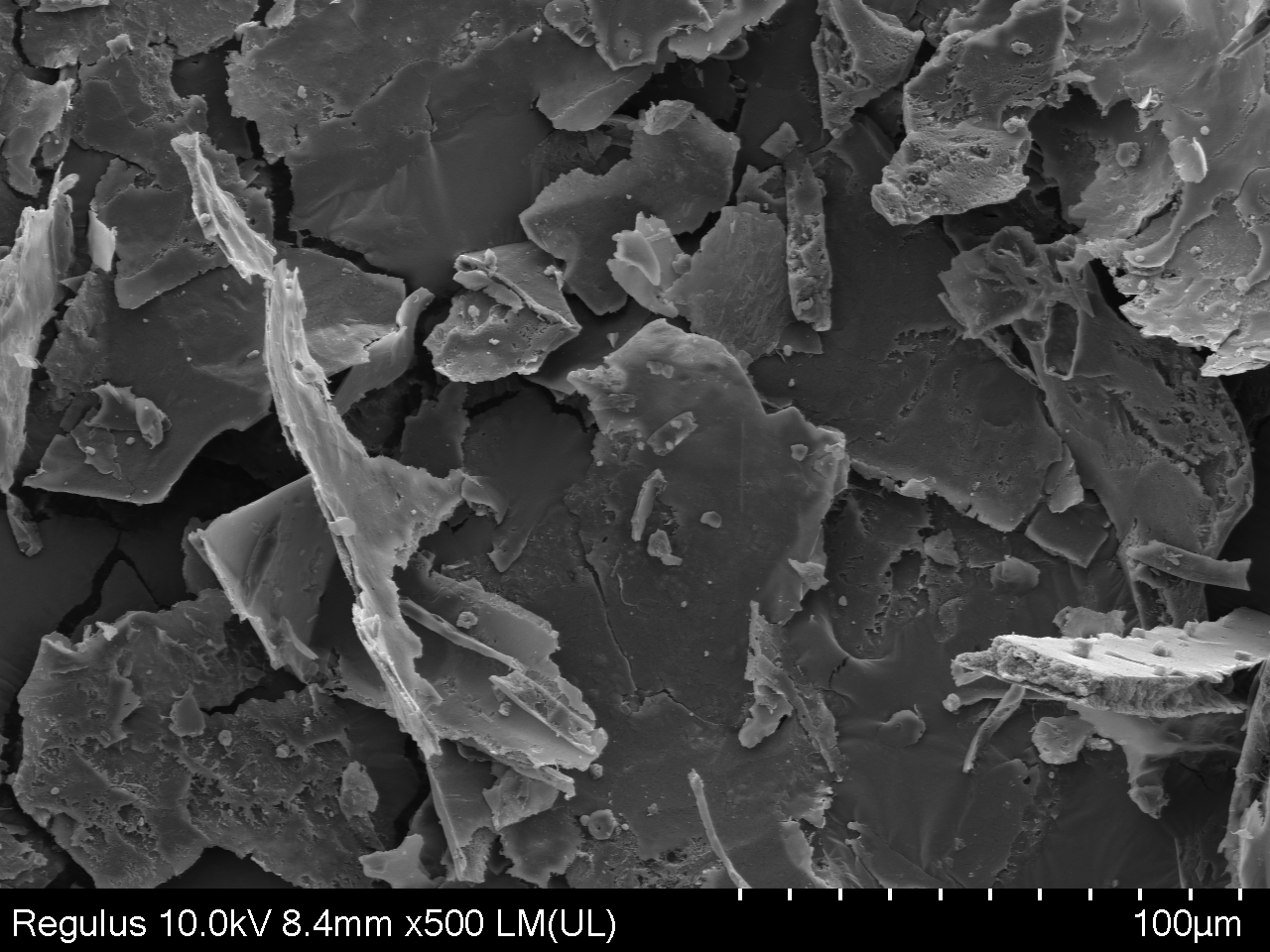

Supplement: Supplementary file 1 [file DataSheet_1.zip › SEM/1_0009.tif]

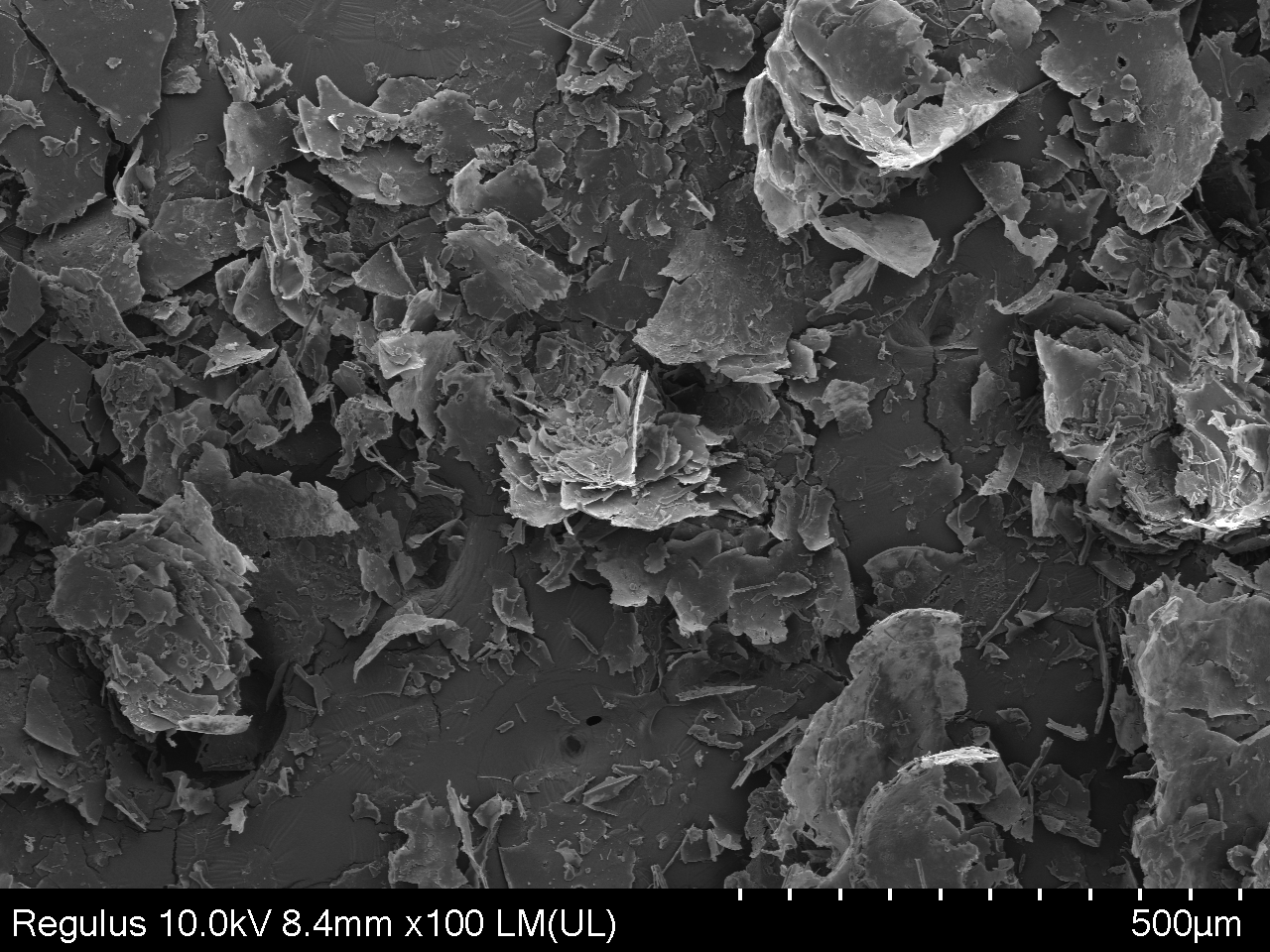

Supplement: Supplementary file 1 [file DataSheet_1.zip › SEM/1_0010.tif]

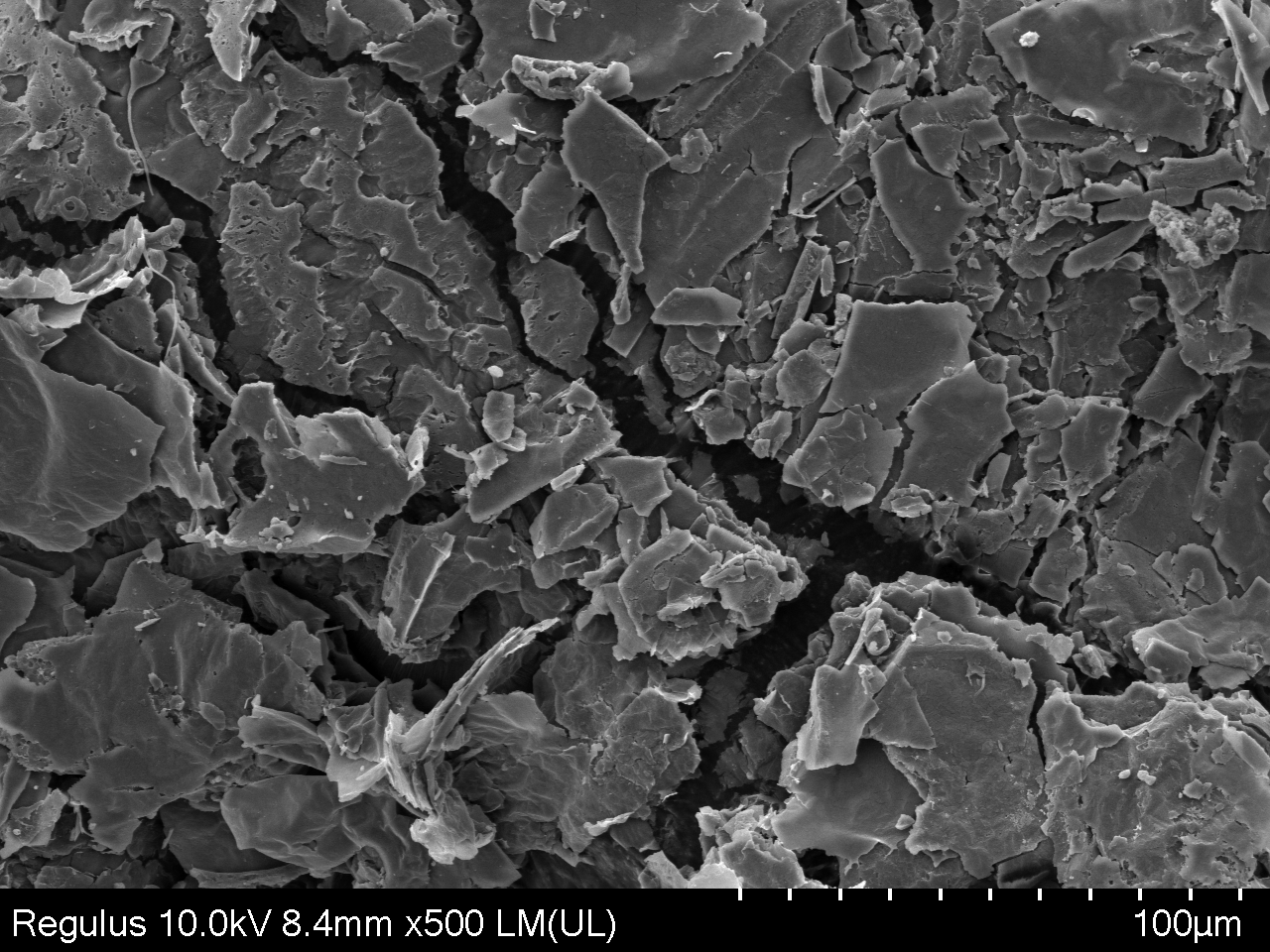

Supplement: Supplementary file 1 [file DataSheet_1.zip › SEM/1_0011.tif]

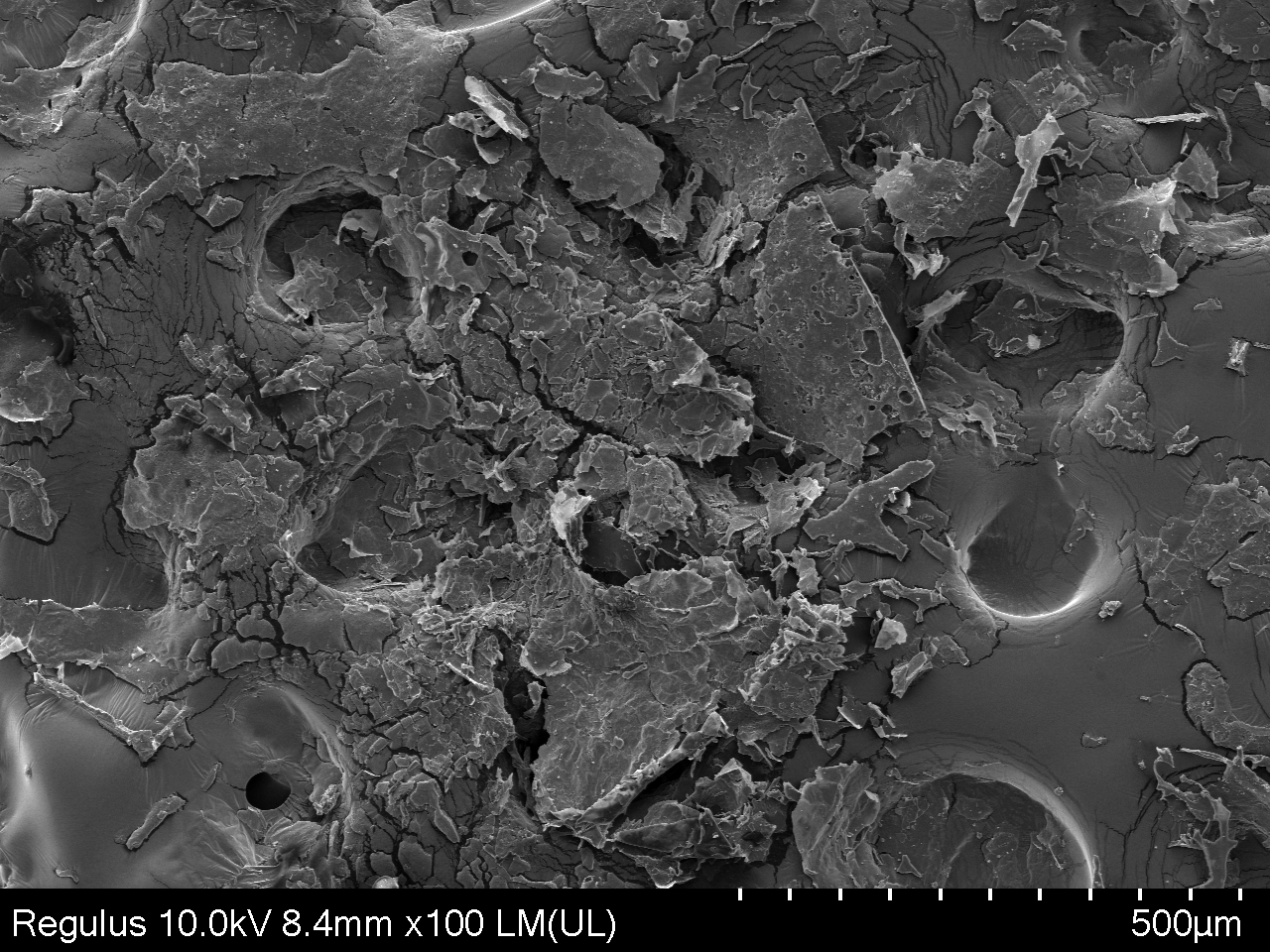

Supplement: Supplementary file 1 [file DataSheet_1.zip › SEM/1_0012.tif]

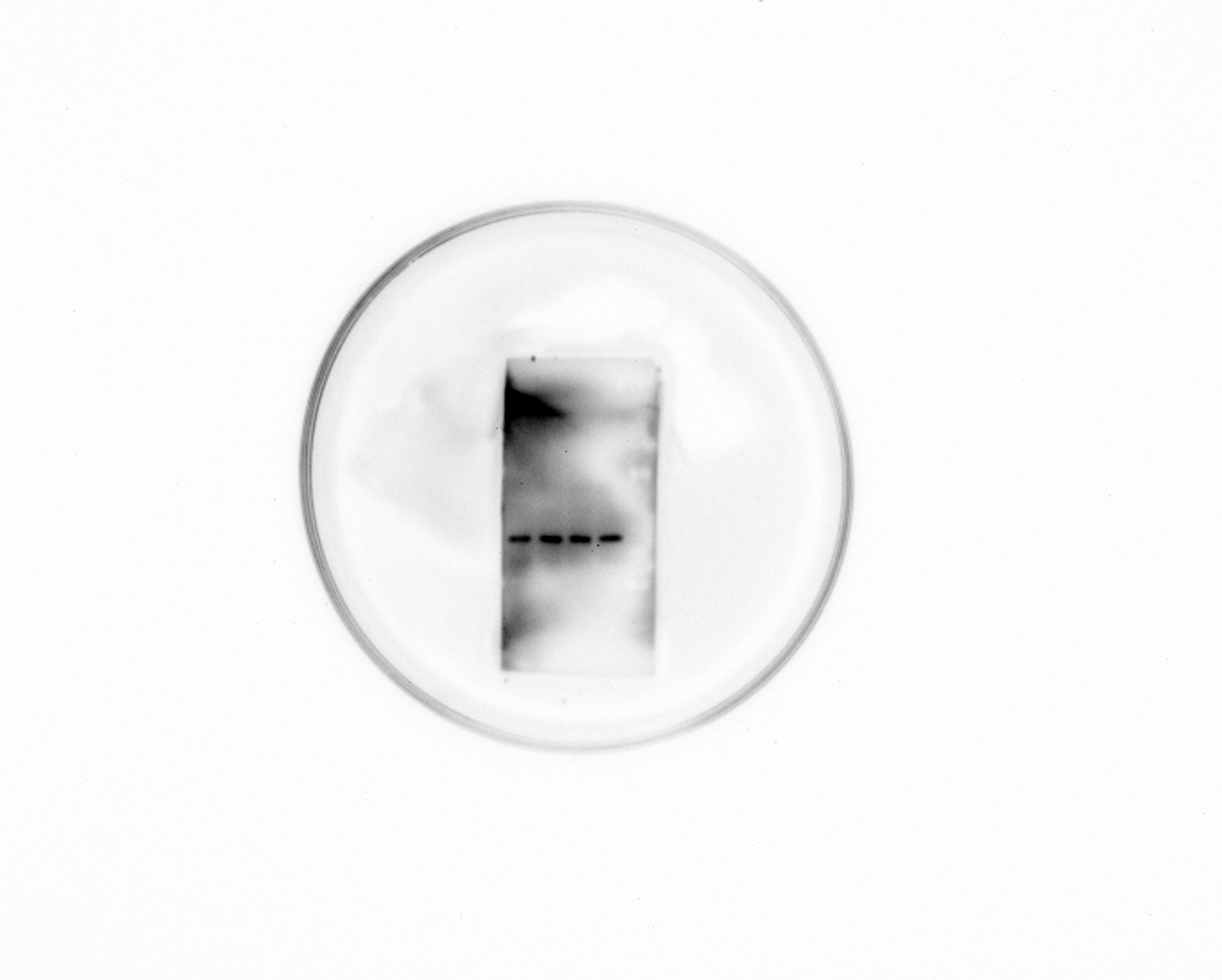

Supplement: Supplementary file 1 [file DataSheet_1.zip › Western Blot/Beclin 1 (3).Tif]

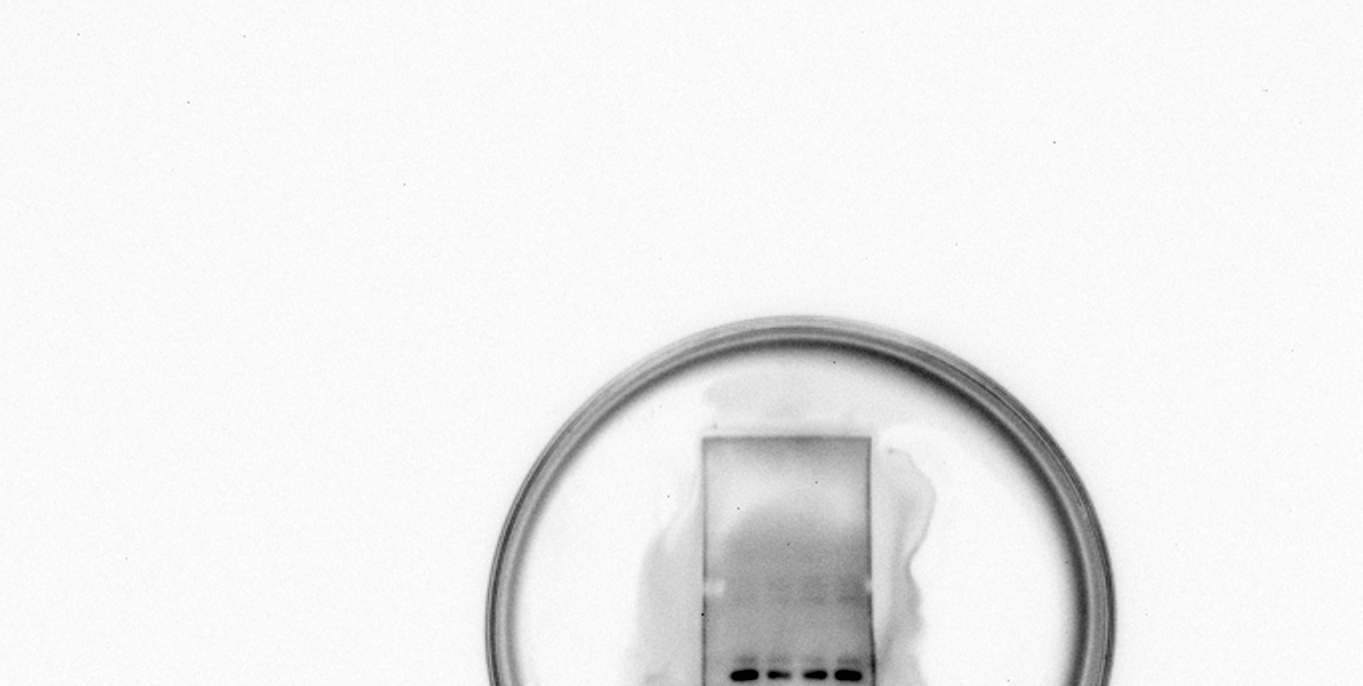

Supplement: Supplementary file 1 [file DataSheet_1.zip › Western Blot/LC3.Tif]

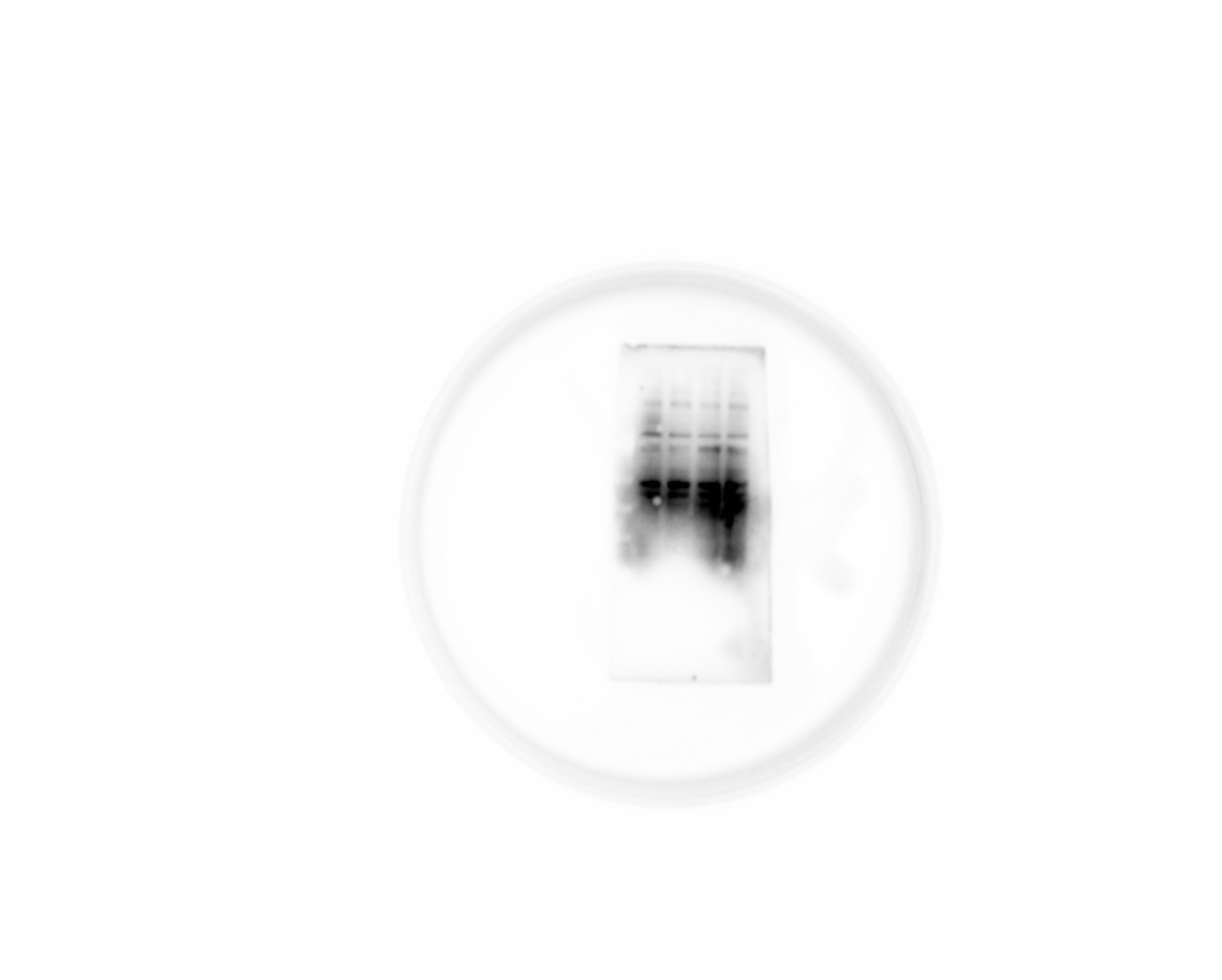

Supplement: Supplementary file 1 [file DataSheet_1.zip › Western Blot/MyD88.Tif]

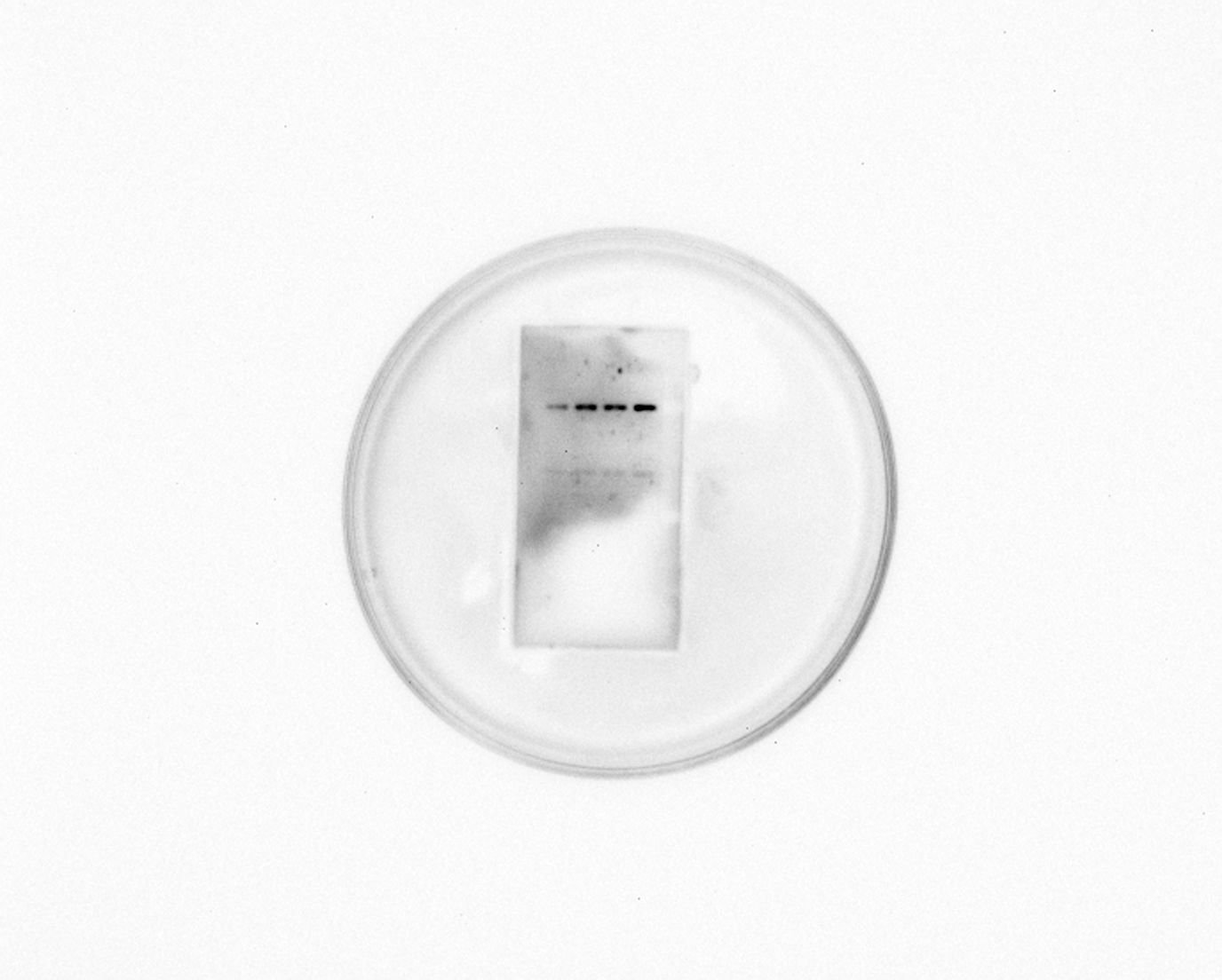

Supplement: Supplementary file 1 [file DataSheet_1.zip › Western Blot/NF-a╩B.Tif]

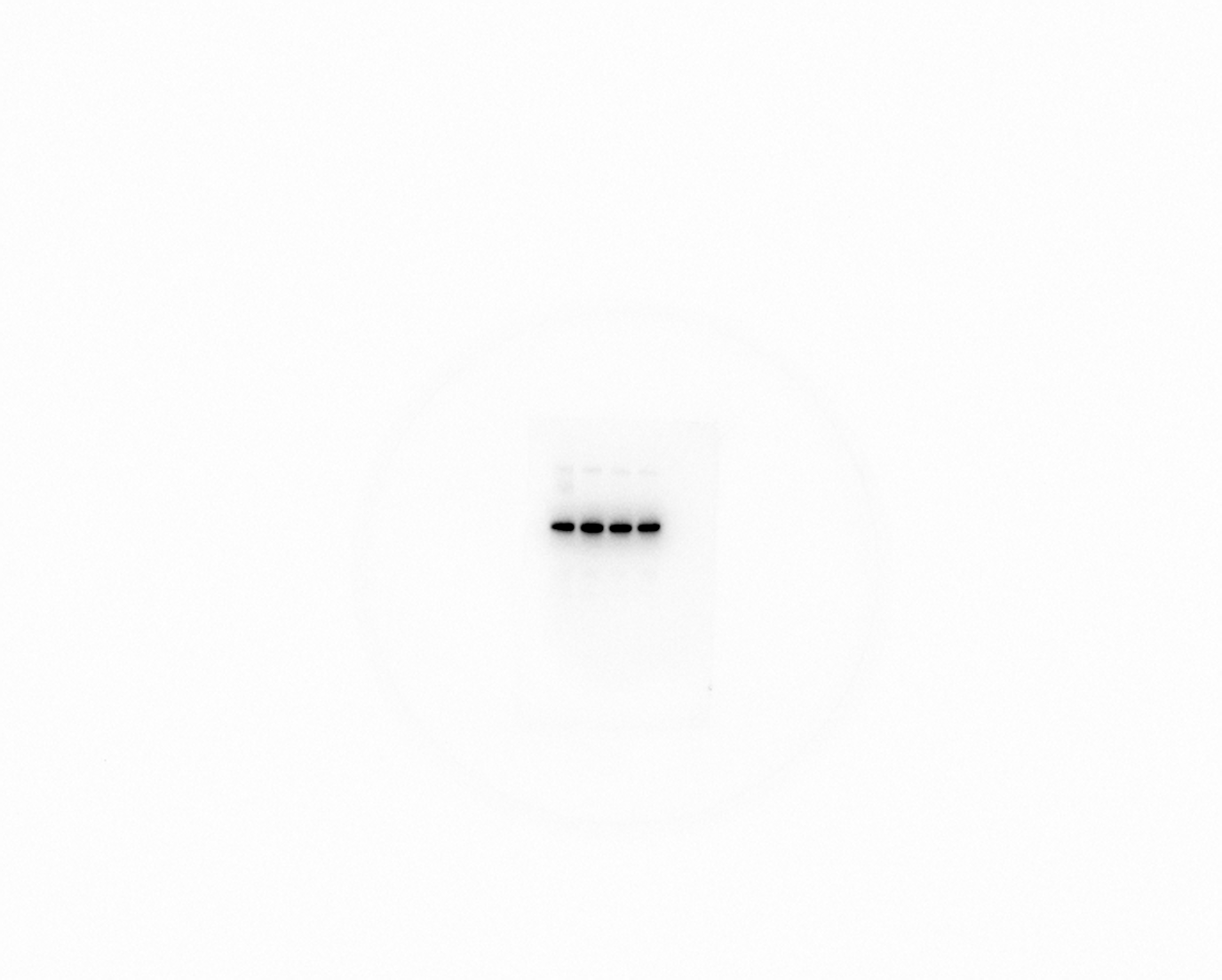

Supplement: Supplementary file 1 [file DataSheet_1.zip › Western Blot/P62.Tif]

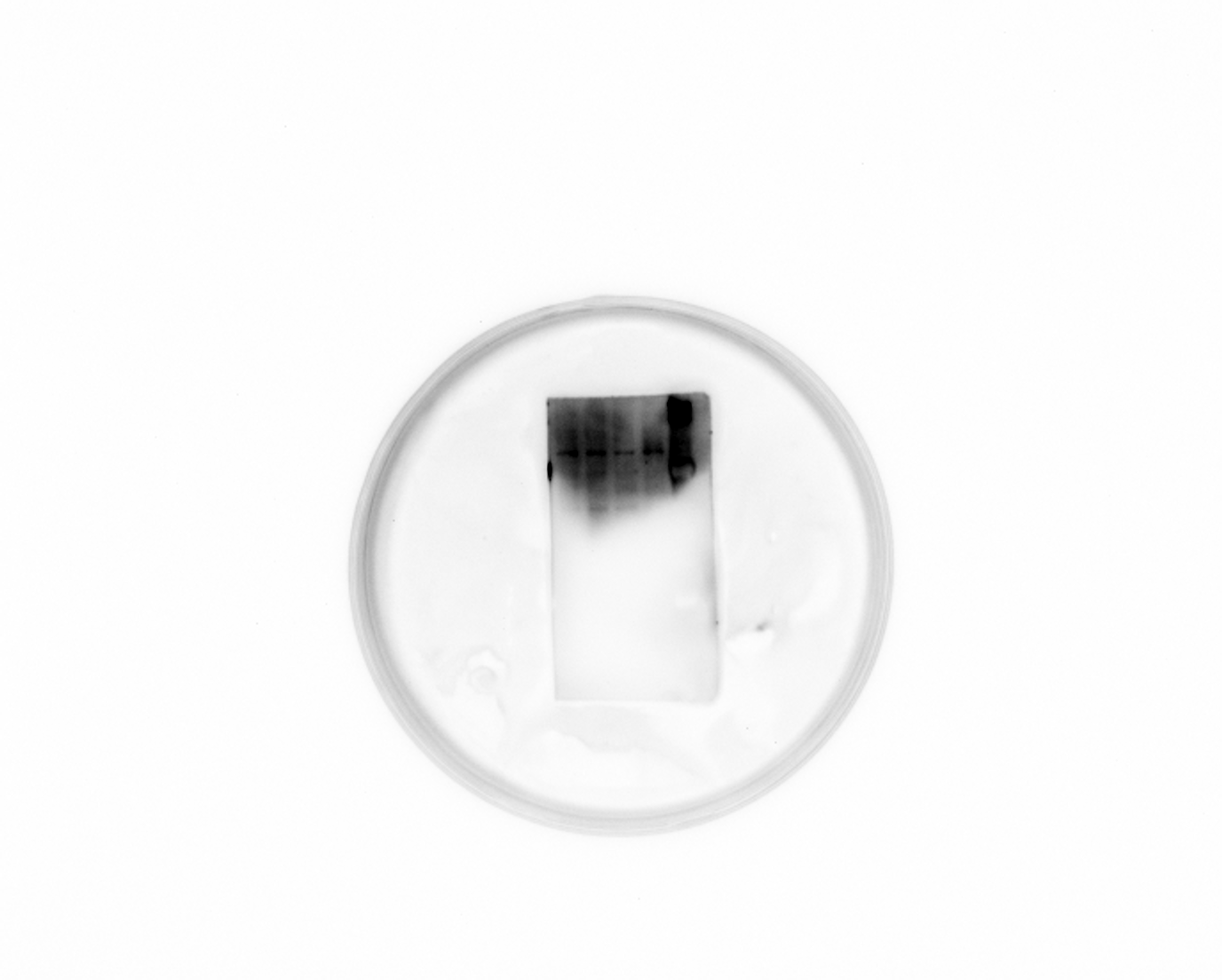

Supplement: Supplementary file 1 [file DataSheet_1.zip › Western Blot/TLR4.Tif]

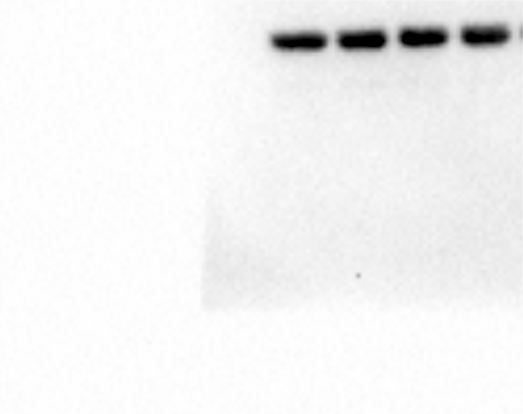

Supplement: Supplementary file 1 [file DataSheet_1.zip › Western Blot/a┬-actin.png]

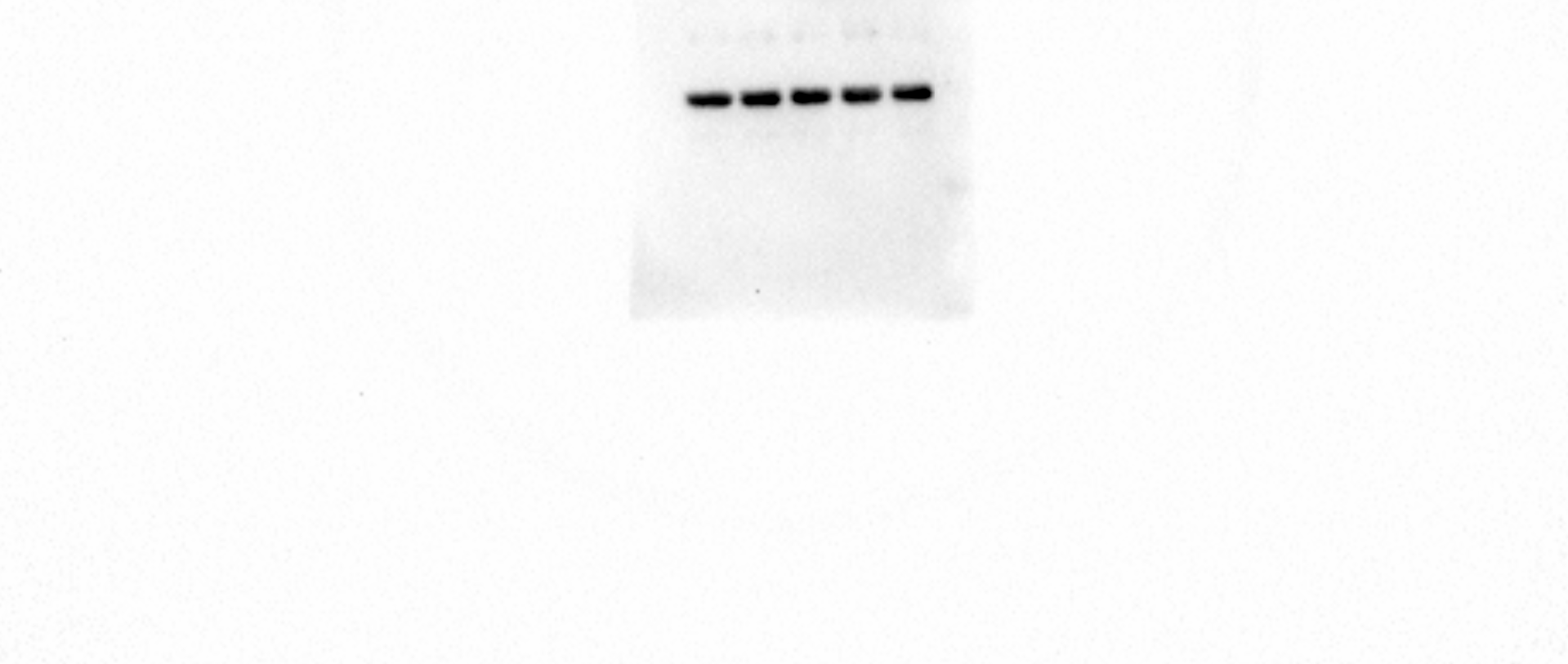

Supplement: Supplementary file 1 [file DataSheet_1.zip › Western Blot/a┬-actin.tif]
